# Microglial burden, activation and dystrophy patterns in frontotemporal lobar degeneration

**DOI:** 10.1186/s12974-020-01907-0

**Published:** 2020-08-10

**Authors:** Ione O. C. Woollacott, Christina E. Toomey, Catherine Strand, Robert Courtney, Bridget C. Benson, Jonathan D. Rohrer, Tammaryn Lashley

**Affiliations:** 1 Dementia Research Centre, Department of Neurodegenerative Disease, UCL Queen Square Institute of Neurology, London, UK; 2 Queen Square Brain Bank for Neurological Disorders, Department of Clinical and Movement Neuroscience, UCL Queen Square Institute of Neurology, 1 Wakefield Street, London, WC1N 1PJ UK; 3 Department of Neurodegenerative Disease, UCL Queen Square Institute of Neurology, London, UK

**Keywords:** Frontotemporal dementia, Frontotemporal lobar degeneration, Microglia, Dystrophy, Neuroinflammation, Progranulin

## Abstract

**Background:**

Microglial dysfunction is implicated in frontotemporal lobar degeneration (FTLD). Although studies have reported excessive microglial activation or senescence (dystrophy) in Alzheimer’s disease (AD), few have explored this in FTLD. We examined regional patterns of microglial burden, activation and dystrophy in sporadic and genetic FTLD, sporadic AD and controls.

**Methods:**

Immunohistochemistry was performed in frontal and temporal grey and white matter from 50 pathologically confirmed FTLD cases (31 sporadic, 19 genetic: 20 FTLD-tau, 26 FTLD-TDP, four FTLD-FUS), five AD cases and five controls, using markers to detect phagocytic (CD68-positive) and antigen-presenting (CR3/43-positive) microglia, and microglia in general (Iba1-positive). Microglial burden and activation (morphology) were assessed quantitatively for each microglial phenotype. Iba1-positive microglia were assessed semi-quantitatively for dystrophy severity and qualitatively for rod-shaped and hypertrophic morphology. Microglia were compared in each region between FTLD, AD and controls, and between different pathological subtypes of FTLD, including its main subtypes (FTLD-tau, FTLD-TDP, FTLD-FUS), and subtypes of FTLD-tau, FTLD-TDP and genetic FTLD. Microglia were also compared between grey and white matter within each lobe for each group.

**Results:**

There was a higher burden of phagocytic and antigen-presenting microglia in FTLD and AD cases than controls, but activation was often not increased. Burden was generally higher in white matter than grey matter, but activation was greater in grey matter. However, microglia varied regionally according to FTLD subtype and disease mechanism. Dystrophy was more severe in FTLD and AD than controls, and more severe in white than grey matter, but this also varied regionally and was particularly extensive in FTLD due to progranulin (*GRN*) mutations. Presence of rod-shaped and hypertrophic microglia also varied by FTLD subtype.

**Conclusions:**

This study demonstrates regionally variable microglial involvement in FTLD and links this to underlying disease mechanisms. This supports investigation of microglial dysfunction in disease models and consideration of anti-senescence therapies in clinical trials.

## Background

Frontotemporal lobar degeneration (FTLD) encompasses a large heterogeneous group of neurodegenerative diseases. FTLD is pathologically diagnosed based on the protein identified in the pathological inclusions, the harbouring cell types and the morphology of the inclusions [1]. The main subtypes of FTLD (FTLD-TDP, FTLD-tau and FTLD-FUS) reflect inclusions of TDP-43, tau and fused in sarcoma (FUS) proteins. FTLD-TDP is further divided into five pathological subtypes, A to E [2], based on the nature of TDP-43 inclusions present. Although FTLD-TDP may be sporadic, mutations in several genes lead to genetic forms of FTLD-TDP, including progranulin (*GRN*), chromosome 9 open reading frame 72 (*C9orf72*) and TANK-binding kinase 1 (*TBK1*), and these dictate the underlying pathology and clinical manifestations. FTLD-tau encompasses genetic cases with tau inclusions due to mutations in microtubule-associated protein tau (*MAPT*) (FTLD-*MAPT*), but also sporadic cases including those with Pick bodies (FTLD-Picks), progressive supranuclear palsy (FTLD-PSP) or corticobasal degeneration (FTLD-CBD). FTLD-FUS cases are rare and contain FUS inclusions [3–5].

There is increasing evidence of microglial dysfunction in the pathogenesis of FTLD [6]. Microglia are the resident innate immune cells of the central nervous system and undergo constant self-renewal, maintaining a steady population throughout life [7]. They exert their functions through diverse phenotypes, reflected in their ability to move between a heterogenous array of morphologies [8–10]. Ramified microglia constantly survey the environment, prune synapses and liaise with other glial cells to support neurons [11]. Following pro-inflammatory signals or insults, ramified microglia respond, and with persistent stimulation they become more activated, rounded and ‘amoeboid’ in shape [8, 10]. Activated microglia migrate towards an insult, proliferate and produce pro-inflammatory cytokines, chemokines and reactive oxygen species, contributing to a state of neuroinflammation [8]. Although this may be initially beneficial in clearing ensuing protein aggregates or dying neurons, over time, chronic activation may damage neurons and synapses, contributing to neurodegeneration. In addition, as microglia undergo repeated self-renewal and telomere shortening throughout the lifetime, they are vulnerable to replicative senescence, becoming increasingly dysfunctional and degenerative with age [9]. Senescent cells are detected histopathologically as dystrophic microglia, which have abnormal morphology: thin, short and few distal branches (deramification), shortened tortuous or beaded cell processes, fragmented cytoplasm, and spheroidal inclusions (rounded swelling) [12]. Dystrophic microglia are seen in the healthy aging brain [13–17]. However, they are seen more commonly in individuals with neurodegenerative disease, especially Alzheimer’s disease (AD) [13, 15, 16, 18–22]. This suggests that by the end stage of disease, many microglia are dysfunctional and senescent, and this may also contribute to neurodegeneration through lack of neuronal support.

Although microglial PET tracers can detect gross patterns of microglial activation in vivo in individuals with symptomatic frontotemporal dementia (FTD) [23–25] and presymptomatic mutation carriers [26, 27], histopathological studies allow more precise examination of regional patterns of microglial burden, and more detailed morphological characterisation to allow appreciation of activation state and dystrophy. The most commonly used microglial markers are cluster of differentiation 68 (CD68), human leukocyte antigen-D related protein-R (HLA-DR), and ionised calcium-binding adapter molecule 1 (Iba1) [28]. CD68 is an intracellular transmembrane bound glycoprotein expressed within the lysosomal, endosomal and plasma membranes of microglia and macrophages and a common marker of activated phagocytic microglia [8, 29]. Major compatibility complex (MHC) class II molecules such as HLA-DR, HLA-DP or HLA-DQ are glycoproteins expressed on the surface of cells with antigen-presenting function. They are mainly expressed on the surface of activated microglia, but allow the detection of microglia with antigen-presenting properties in ramified and activated states [12, 15, 30]. Iba1 is an intracellular calcium-binding protein constitutively expressed within the cytoplasm of ramified microglia and upregulated on activated microglia, so it detects microglia in all activation states and is also very useful for examining cell morphology to detect dystrophy [10, 16, 31, 32].

Early studies described extensive microgliosis in FTLD [33–38]. However, only one study has explored microglia across the spectrum of pathologically diagnosed FTLD, performing a semi-quantitative assessment of the frequency and activation state of CD68-positive microglia in frontal and temporal cortical grey and subcortical white matter [39]. This suggested that there are more numerous and/or more activated microglia in FTLD than controls, particularly in frontal white matter, although this varied by pathological subtype. Several histological studies have examined microglia in specific subtypes of FTLD, particularly FTLD-TDP [23, 40–44]. These suggest that microglia are altered in FTLD-TDP in a regionally selective manner, particularly in white matter [40, 41, 43], but whether microglia vary across all FTLD pathological subtypes has not been explored. As regional pathology [1, 45, 46] and grey and white matter neuroimaging abnormalities [47–52] vary across the spectrum of genetic FTD, microglia may also differ regionally between genetic FTLD subtypes due to different mutations. One study has compared microglia between the three main genetic subtypes, showing that FTLD-*MAPT* cases had more numerous and/or more activated CD68-positive microglia in temporal white matter compared with FTLD-*GRN*, FTLD-*C9orf72* and sporadic FTLD cases [39]. A more recent study of FTLD-*GRN* and FTLD-*C9orf72* cases and controls found that the burden and morphology of CD68- and Iba1-positive microglia varied regionally depending on the mutation [53]. Dystrophic microglia have only been identified in small studies of certain subtypes of FTLD, particularly FTLD-TDPA, and these also vary regionally [42, 53, 54]. Unusual microglial morphologies such as thin, elongated cells known as rod-shaped microglia, and also hypertrophic microglia, which have short, thickened, bushy processes, are present in AD cases [13, 55], and rod-shaped microglia have been noted in FTLD-TDPA due to *GRN* or *C9orf72* mutations [42, 53] and sporadic FTLD-TDPA cases [42]. However, microglial dystrophy and rod-shaped or hypertrophic microglia have not been explored across the full spectrum of FTLD.

In this study, we compared microglial burden, activation and dystrophy in the major FTLD pathological subtypes, compared with AD cases and neuropathologically normal controls, and examined for presence of rod-shaped and hypertrophic microglia in each group. We hypothesised that microglial burden and activation state, but also microglial dystrophy and presence of microglia with unusual morphologies, would be increased in FTLD, but that this may vary regionally according to FTLD subtype and disease mechanism, including underlying mutation.

## Methods

### Cases

**Fig. 1:**
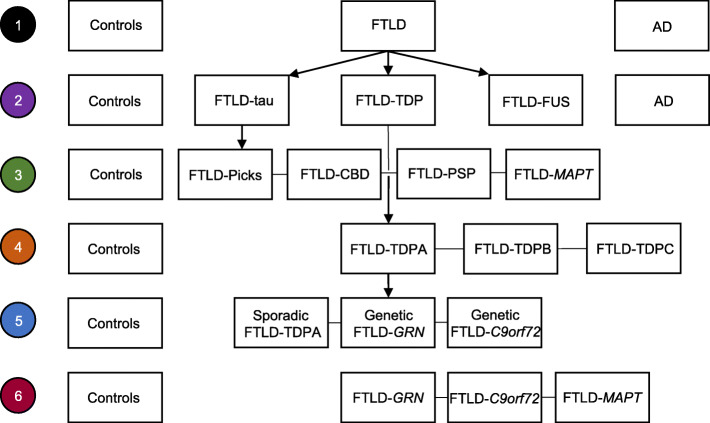
Approach to group comparisons. Demographics and microglial parameters (burden, circularity and perimeter values and dystrophy scores) were compared between groups using six levels of comparison (numbers in circles denote level): 1: overall disease groups; 2: controls, main FTLD subtypes (FTLD-TDP, FTLD-tau and FTLD-FUS) and AD; 3: controls and FTLD-tau subtypes; 4: controls and FTLD-TDP subtypes; 5: controls and either sporadic or genetic FTLD-TDPA (genetic had either *GRN* mutations, FTLD-*GRN*, or *C9orf72* expansions, FTLD-*C9orf72*); 6: controls and genetic FTLD subtypes (the FTLD-*C9orf72* group included only FTLD-TDPA cases to ensure comparison of mutation rather than pathological subtype)

Sixty cases (50 FTLD, five sporadic AD, five controls without neurodegenerative pathology) were selected for analysis from the archives at Queen Square Brain Bank for Neurological Disorders (QSBB) and the MRC London Neurodegenerative Diseases Brain Bank, Institute of Psychiatry, King’s College London. Case demographics and pathological subtypes are summarised in Table 1 and detailed in Supplementary Table 1, Additional File 1. The final FTLD cohort included the following: 31 sporadic and 19 genetic FTLD cases: 26 FTLD-TDP (16 FTLD-TDPA [five sporadic, 11 genetic], five FTLD-TDPB [two sporadic, three genetic] and five FTLD-TDPC), 20 FTLD-tau (five each of FTLD-CBD, FTLD-*MAPT*, FTLD-Picks and FTLD-PSP) and four FTLD-FUS. The 19 genetic cases included five with *GRN* mutations (FTLD-*GRN*; four C31fs and one Q130fs(388_391delCAGT); all FTLD-TDPA), five with +16 splice site mutations on the intron to exon 10 (10 + 16 mutations) in *MAPT* (FTLD-*MAPT*), eight with the *C9orf72* expansion (FTLD-*C9orf72*; five with FTLD-TDPA and three with FTLD-TDPB) and one with a *TBK1* A705fs mutation (FTLD-TDPA)**.** Microglial parameters and dystrophy scores were compared between disease groups and controls, and between different subtypes of FTLD as shown in Fig. 1, as well as between grey and white matter within each lobe in each group.

**Table 1:** Demographics of cases and controls

Pathological diagnosis	*N*	Genetic mutation (*N*)	Clinical diagnosis (*N*)	Sex (M/F)	Mean (range) age at symptom onset in years	Mean (range) age at death in years	Mean (range) disease duration in years	Mean (range) post-mortem delay (hours)
**Controls**	5	n/a	Control	1/4	n/a	67.4 (38–80)	n/a	45.7 (24.0–80.6)
**AD**	5	n/a	Amnestic AD	3/2	58.8 (49–65)	69.2 (62.0–73.0)	10.4 (5.0–17.0)	45.8 (31.1–76.7)
**FTLD**	50	Sporadic = 31 Genetic = 19: *GRN* = 5 *C9orf72* = 8 *MAPT* = 5 *TBK1* = 1	bvFTD = 23 nfvPPA = 8 FTD-MND = 8 svPPA = 5 PSPS = 5 CBS = 1	29/21	57.8 (37–66)	66.5 (51.3–84.0)	8.7 (2.1–18.7)	60.7 (10.8–157.6)
**FTLD subgroups**								
**FTLD-tau**	20	Sporadic = 15 Genetic = 5 (*MAPT*)	bvFTD = 10 nfvPPA = 4 PSPS = 5 CBS = 1	13/7	58.2 (37–71)	68.1 (52.4–84.0)	9.9 (5.5–16.4)	54.9 (24.0–103.3)
FTLD-CBD	5	All sporadic	nfvPPA = 3 PSPS =1 CBS = 1	2/3	60.2 (57–65)	69.0 (64.8–73.8)	8.8 (5.5–11.1)	68.9 (37.2–103.3)
FTLD-*MAPT*	5	Genetic = 5 (all *MAPT* 10 + 16)	bvFTD = 5	2/3	48.0 (37–58)	59.6 (52.4–68.4)	11.6 (8.1–16.4)	45.9 (24.0–64.0)
FTLD-Picks	5	All sporadic	bvFTD = 5	5/0	57.6 (52–64)	68.1 (63.5–75.6)	10.5 (5.5–15.6)	47.8 (24.0–94.7)
FTLD-PSP	5	All sporadic	PSPS = 4 nfvPPA = 1	4/1	67.0 (62–71)	75.6 (68.0–84.0)	8.6 (6.0–13.0)	57.2 (25.5–86.4)
**FTLD-TDP**	26	Sporadic = 12 Genetic = 14 *C9orf72* = 8 *GRN* = 5 *TBK1* = 1	bvFTD = 9 nfvPPA = 4 FTD-MND = 8 svPPA = 5	12/14	59.4 (50–76)	67.2 (53.1–78.6)	7.8 (2.1–18.7)	68.8 * (10.8–157.6)
FTLD-TDPA	16	Sporadic = 5 Genetic = 11 *C9orf72* = 5 *GRN* = 5 *TBK1* = 1	bvFTD = 7 nfvPPA = 4 FTD-MND = 5	7/9	60.6 (50–76)	67.0 (53.1–78.6)	6.4 (2.1–10.3)	77.3 (29.3–157.6)
FTLD-TDPB	5	Sporadic = 2 Genetic = 3 (*C9orf72*)	bvFTD = 2 FTD-MND = 3	2/3	56.2 (50–63)	62.1 (56.2–67.2)	5.9 (4.2–8.1)	68.5 (10.8–96.0)
FTLD-TDPC	5	All sporadic	svPPA = 5	3/2	58.8 (52–64)	73.0 (65.4–78.6)	14.2 (10.3–18.7)	43.8 (19.0–83.7)
**FTLD-FUS** (all aFTLD-U)	4	All sporadic	bvFTD = 4	4/0	45.4 (40–51)	53.9 51.3–60.4	8.4 (5.5–11.3)	39.0 (12.0–72.0)
**FTLD cases used for genetic group analysis** (all included in groups above)	15	*GRN* = 5 (all FTLD-TDPA) *MAPT* = 5 (all FTLD-*MAPT*) *C9orf72* = 5 (all FTLD-TDPA)	bvFTD = 4 nfvPPA = 1 bvFTD = 5 FTD-MND = 3 nfvPPA = 2	2/3 2/3 1/4	58.0 (62–67) 48.0 (37–58) 60.8 (55–68)	64.6 (55.3–74.2) 59.6 (52.4–68.4) 68.6 (62.7–75.1)	6.6 (5.3–8.4) 11.6 (8.1–16.4) 7.8 (5.7–10.3)	83.1 (29.3–157.6) 45.9 (24.0–64.0) 78.7 (51.9–107.1)

Summary of demographics of FTLD and AD cases and controls analysed. *N* number of cases, *M* male, *F* female

### Immunohistochemistry

**Fig. 2:**
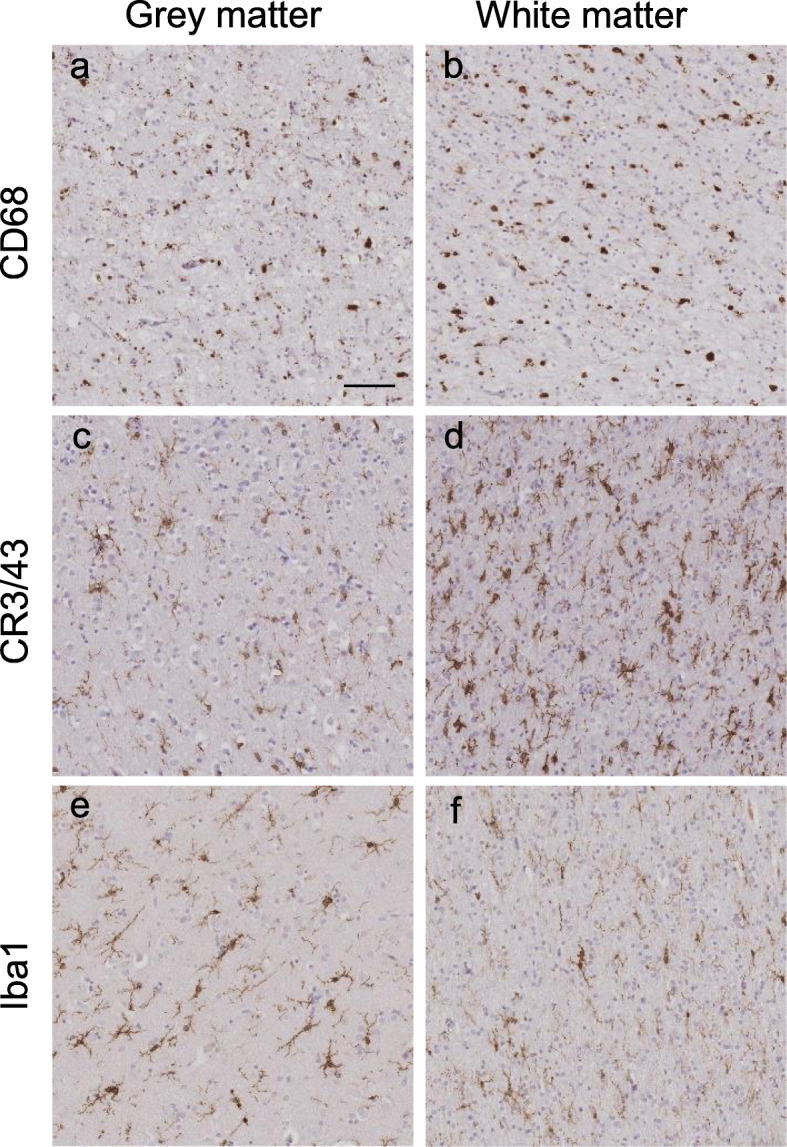
Immunohistochemical staining of each microglial marker. Representative images of immunohistochemical staining in cortical grey matter (**a, c, e**) and subcortical white matter (**b, d, f**) of sections with CD68-positive microglia (**a, b**) CR3/43-positive microglia (**c, d**) and Iba1-positive microglia (**e, f**). Images were taken from the following cases (in Supplementary Table 1): **a** and **b** from case 25; **c–f** from case 55. Scale bar represents 50 μm in all images

The left anterior frontal lobe and left temporal lobe were analysed, split into four regions: frontal grey (FG), frontal white (FW), temporal grey (TG) and temporal white (TW) matter. Analysis of grey matter included all cortical layers and white matter included subcortical white matter only. Immunohistochemistry was performed using antibodies to detect three microglial markers: CD68, CR3/43 (which detects HLA-DP/DQ/DR) and Iba1 on sequential sections for each case (Fig. 2). Briefly, 8-μm paraffin embedded sections were cut and dried, de-paraffinised in xylene and rehydrated. Endogenous peroxidase activity was blocked in 0.3% H_2_O_2_/methanol solution and pre-treated by pressure cooking sections in citrate buffer pH 6.0. Non-specific protein binding was blocked in 10% non-fat milk/TBS. The primary antibodies (CD68, Dako 1:100; CR3/43, Dako 1:150; Iba1, Wako Chemicals 1:1000) were applied and sections incubated for 60 min at room temperature. Sections were incubated in the relevant biotinylated secondary antibody (biotinylated rabbit anti-mouse or biotinylated swine anti-mouse, Dako 1:200) for 30 min at room temperature before incubating in Avidin-Biotin Complex solution (Vector Laboratories Inc.). 3,3′-Di-aminobenzidine (DAB) was used as the chromogen, and sections were counterstained in Mayer’s haematoxylin solution.

### Analysis of microglial burden and activation

The sequential sections stained for each of the three microglial markers were digitally scanned using a Leica SCN400F scanner at × 40 magnification. Images were loaded into Aperio ImageScope software and identical areas of interest for analysis were identified within each of the four regions (FG, FW, TG and TW) for each case. A macro using Aperio ImageScope, ImageJ and Python programming software was used to identify and quantitatively analyse microglia in each region. Ten random squares (each 1000 by 1000 pixels, equivalent to 500 μm^2^) were generated and analysed in the area of interest within each region for each case. ImageJ was used to identify all DAB staining for each marker, employing a pre-set colour threshold, with a hue of 0 to 30 (for DAB detection) and a saturation of between 60 and 80 (adjusted according to background staining level). This was to ensure selection of only DAB stained cells rather than weaker, non-specific staining. A pre-set diameter of ten pixels (5 μm) was used to exclude selection of any stained cells smaller than this, in order to minimise inclusion of parts of microglia that were predominantly on a different plane. Microglial burden was quantified using the measure function in ImageJ to quantify the percentage area (areal fraction) stained with DAB. Microglial morphology was analysed using the Hull and Circle function to quantify the circularity (cell shape, with 0 representing an imperfect shape (ramified) and a score closer to 1 a perfect circle (amoeboid)) and perimeter (cell size, with a smaller perimeter indicating less ramified microglia and a larger perimeter indicating more ramified microglia) of each stained cell present. Circularity and perimeter values were averaged across all cells present. Percentage area, circularity and perimeter values were then averaged across the ten squares to give mean values in each region. This was performed for each of the three markers for every case.

### Analysis of microglial dystrophy

**Fig. 3:**
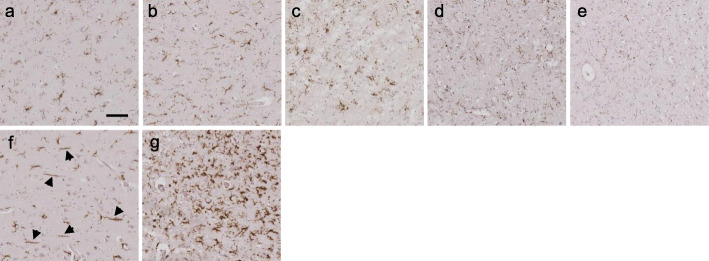
Scoring of the severity of microglial dystrophy and examples of rod-shaped and hypertrophic microglia. Iba1 immunohistochemical staining showing representative images of the semi-quantitative analysis of microglial dystrophy and the different microglial morphologies. Grey matter sections from a healthy control (**a**, case 2 in Supplementary Table 1) and four different FTLD cases (in Supplementary Table 1: **b** case 44; **c** case 22; **d** case 42; **e** case 37) show appearances of dystrophic microglia, graded in severity, from **a** (1 = no dystrophy), **b** (2 = mild dystrophy), **c** (3 = moderate), **d** (4 = severe), to **e** (5 = very severe). When dystrophy is very severe, minimal staining is visible due to complete disruption of normal cell structure. **f** Several rod-shaped microglia in the frontal grey matter of an FTLD-TDPB case with the *C9orf72* expansion (case 48; black arrows point to these cells). **g** Multiple hypertrophic microglia throughout the frontal grey matter of an FTLD-Picks case (case 25; most cells present have a bushy appearance with short, thick processes). Scale bar represents 50 μm in all images

Microglial dystrophy was assessed on the Iba1-stained slides from the ten random squares previously generated from each region. Dystrophy was determined as the loss of fine branches (deramification), or thin, shortened, beaded, tortuous or fragmented processes or cytoplasm, or all of these changes [12, 13]. The severity of dystrophy was assessed using a semi-quantitative, ordinal scoring system, adapted from the semi-quantitative scoring system used in assessments of the burden and activation of microglia in other studies of FTLD [39, 44]. Scores were as follows: 1 = no dystrophy: microglia were of normal morphology (ramified, amoeboid or hypertrophic); 2 = mild dystrophy: some dystrophic microglia seen, but at least half present were of non-dystrophic morphology; 3 = moderate dystrophy: most microglia had dystrophic morphology, but some were of non-dystrophic, normal morphology; 4 = severe dystrophy: all microglia had dystrophic morphology, but some cell bodies or processes were still visible; 5 = very severe dystrophy: all microglia were severely dystrophic, with few or no intact cell bodies or processes, or only generalised punctate staining present. Each case was assigned an overall ‘dystrophy score’ for each region, which was used in group analyses. Examples of sections containing microglia with each dystrophy score are shown in Fig. 3a–e.

### Evaluation of rod-shaped and hypertrophic microglia

Presence of any rod-shaped microglia (Fig. 3f) or hypertrophic microglia (Fig. 3g) was noted qualitatively in each region for every case using visual assessment of the ten randomly selected squares (generated within each region as described above) from Iba1-stained sections at × 40 magnification. The frequency of cells with these morphologies was not quantified given the exploratory nature of this part of the study; if at least one cell with this morphology was visible in at least one of the ten squares, this was recorded as being present, but if many were present this was also noted.

### Statistical analysis

A group comparison analysis was used to compare microglial parameters (burden, circularity and perimeter) for the three different microglial markers, and dystrophy scores (for Iba1-positive microglia) in each region (FG, FW, TG and TW) in a hierarchical manner based on underlying pathological diagnosis. Microglial parameters and dystrophy scores were compared between groups using a stepwise, multi-level approach as detailed in Fig. 1. This was performed to allow appreciation of how microglia differed between overall disease groups (FTLD, AD and controls) and between the various subtypes of FTLD. In addition, microglial parameters and dystrophy scores were compared between grey and white matter within each lobe (FG versus FW and TG versus TW) for each group. Comparisons between groups were performed using non-parametric tests given the small size of some groups (Kruskal Wallis tests followed by Dunn’s test for post hoc comparisons). Comparisons between grey and white matter within each group were performed using Wilcoxon signed rank tests for paired comparisons. Demographics including age at death (AAD), age at onset of symptoms (AAO), disease duration and post-mortem delay were also compared between groups as detailed in Fig. 1, using non-parametric tests (rank sum tests for two groups or Kruskal Wallis with Dunn’s test for post hoc comparisons for more than two groups). Sex was compared between groups using Fisher’s exact tests. STATA version 14.2 was used to analyse data, with a significance threshold of *p* < 0.05 and confidence interval of 95% for all statistical tests. Graphs of comparisons of microglial parameters between groups and between grey and white matter were produced in GraphPad Prism version 7. Heat maps of *p* values for all comparisons (Supplementary Figs. 4 to 7, Additional File 2) were produced in Microsoft Excel.

## Results

**Fig. 4:**
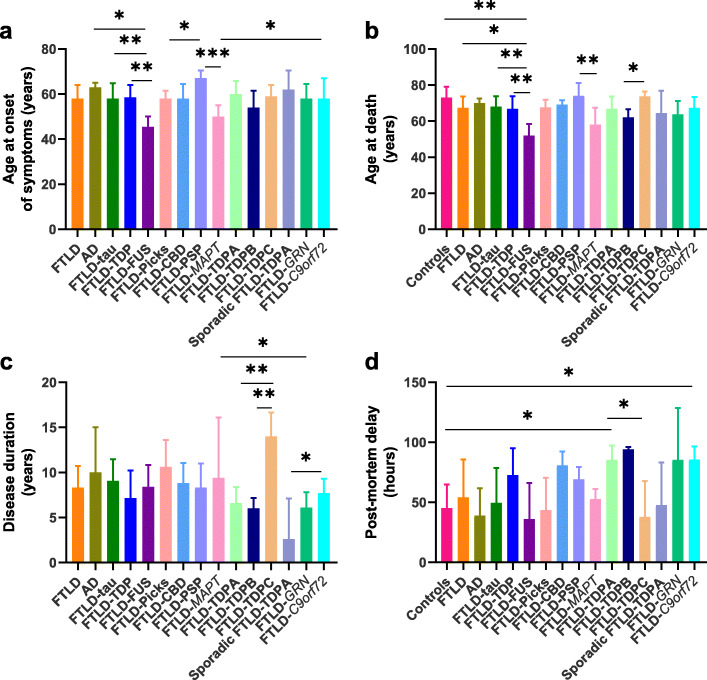
Comparison of demographics between groups. Graphs show comparisons of age at onset (AAO) (**a**), age at death (AAD) (**b**), disease duration (**c**) and post-mortem delay (**d**) across groups. Bars show medians and error bars represent interquartile ranges. *p < 0.05; **p < 0.01; ***p ≤ 0.001; ****p ≤ 0.0001

Demographics for each group are summarised in Table 1 and full details are in Supplementary Table 1, Additional File 1. Comparisons of AAO, AAD, disease duration and post-mortem delay across groups are shown in Fig. 4. Sex, AAO, AAD and post-mortem delay were generally similar between cases and controls or pathological subtypes, with a few exceptions. FTLD-FUS cases had an earlier AAO than FTLD-TDP (*p =* 0.004), FTLD-tau (*p* = 0.006) and AD (*p =* 0.022) cases (Fig. 4a). AAO differed across FTLD-tau subtypes (*p =* 0.005): FTLD-PSP cases had a later AAO than FTLD-Picks (*p =* 0.034) and FTLD-*MAPT* (*p =* 0.0004) cases. AAO did not differ across FTLD-TDP subtypes or between sporadic and genetic FTLD-TDPA cases. However, FTLD-*MAPT* cases had an earlier AAO than FTLD-*C9orf72* cases (*p =* 0.025). FTLD-FUS cases had a younger AAD compared with controls (*p* = 0.006), FTLD-TDP (*p* = 0.008), FTLD-tau (*p* = 0.004) and AD (*p* = 0.012) cases (Fig. 4b). FTLD-*MAPT* cases had a younger AAD than FTLD-PSP cases (*p =* 0.004). FTLD-TDPC cases had an older AAD than FTLD-TDPB cases (*p* = 0.022). FTLD-TDPC cases had a longer disease duration than FTLD-TDPA (*p =* 0.002) and FTLD-TDPB (*p =* 0.004) cases (Fig. 4c). Disease duration was longer for FTLD-TDPA cases with *C9orf72* expansions than sporadic FTLD-TDPA cases (*p =* 0.04), and for FTLD-*MAPT* than FTLD-*GRN* cases (*p* = 0.011). FTLD-TDPA cases had a longer post-mortem delay than FTLD-TDPC cases (*p* = 0.033) and compared with controls (*p* = 0.046), and FTLD-*C9orf72* cases had a longer post-mortem delay than controls (*p* = 0.039) (Fig. 4d).

All *p* values obtained from comparisons of microglial burden, circularity and perimeter between groups and between grey and white matter within each group are detailed in Supplementary Figs.4 to 6 in Additional File 2 as heat maps. All *p* values obtained from comparisons of dystrophy scores between groups and between grey and white matter within each group are detailed in Supplementary Fig. 7 in Additional File 2 as a heat map.

### Microglial burden

#### Neurodegenerative disease comparisons

**Fig. 5:**
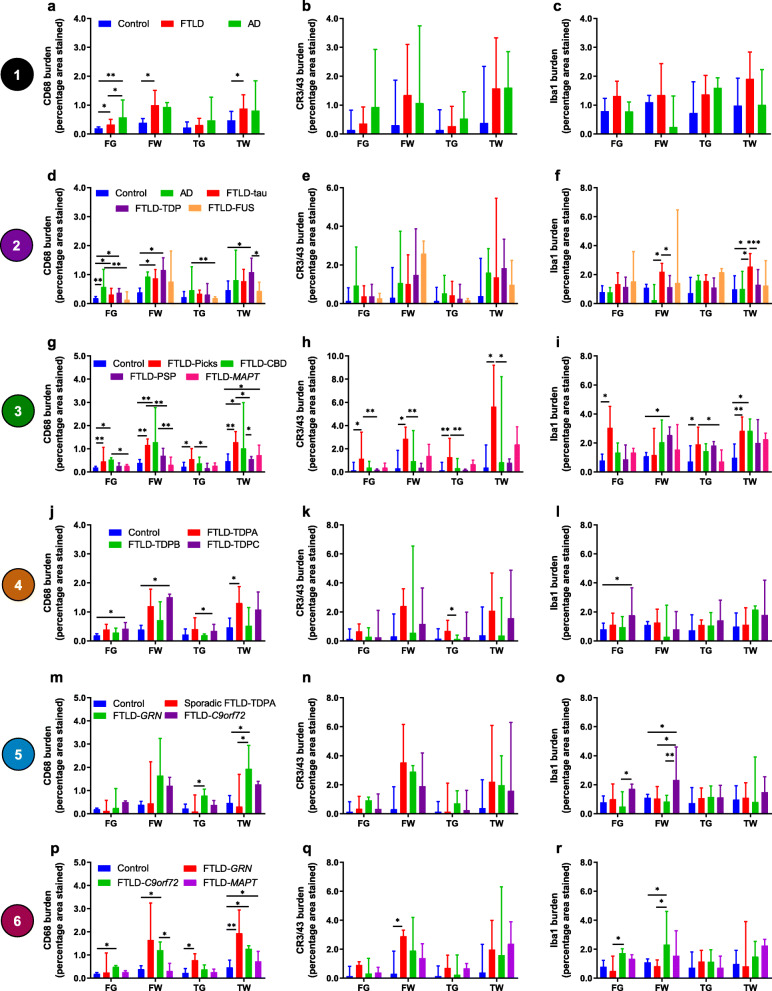
Microglial burden compared between groups in each region. Comparisons of the burden of CD68-positive (**a, d, g, j, m, p**), CR3/43-positive (**b, e, h, k, n, q**) and Iba1-positive (**c, f, i, l, o, r**) microglia for each group comparison level shown within Fig. 1 (numbers in coloured circles on the left represent level of comparison). Graphs show median microglial burden (percentage area values) in each brain region: frontal grey (FG), frontal white (FW), temporal grey (TG) and temporal white (TW) matter. See legend in first graph on each row for bar colours. Error bars represent interquartile range. *p < 0.05; **p < 0.01; ***p ≤ 0.001; ****p ≤ 0.0001

FTLD cases had more CD68-positive microglia in FG, FW and TW compared with controls (Fig. 5a). AD cases had a higher burden in FG compared with controls and FTLD cases. The burden of CR3/43-positive and Iba1-positive microglia was highly variable across cases within each group and comparisons between groups did not reach significance in any region (Fig. 5b, c). Grey versus white matter comparisons revealed that the burden of CD68- and CR3/43-positive microglia was generally higher in white matter than grey matter for all groups (Supplementary Fig. 1a, b). For the frontal lobe, values were significantly higher in white matter for FTLD cases and controls, but not for AD cases. For the temporal lobe, values were significantly higher in white matter for all three groups, particularly for FTLD but less so for AD and controls. For Iba1-positive microglia, the only significant difference was a higher burden of microglia in TW compared with TG in FTLD cases (Supplementary Fig. 1c).

#### Main FTLD subtypes

FTLD-tau and FTLD-TDP cases had a higher burden of CD68-positive microglia in FG and FW compared with controls, but there was a similar burden in each subtype in all regions. FTLD-TDP cases also had a higher burden in TW compared with controls and FTLD-FUS cases (Fig. 5d). In contrast, the burden of CD68-positive microglia in FTLD-FUS cases was low, similar to controls in all regions. AD cases had a higher burden of CD68-positive microglia in FG compared with controls and in FG and TG compared with FTLD-FUS cases. AD and FTLD-tau cases had a similar burden of this phenotype in all regions (Fig. 5d). There were no significant differences in the burden of CR3/43-positive microglia between any group (Fig. 5e). FTLD-tau cases had a particularly high burden of Iba1-positive microglia in white matter, with more in both FW and TW compared with FTLD-TDP and AD cases, but only in TW compared with controls (Fig. 5f). However, FTLD-TDP and FTLD-FUS cases had a similar burden of Iba1-positive microglia to controls in all regions. Grey versus white matter comparisons showed a significant difference in the burden of CD68- and CR3/43-positive microglia between grey and white matter in frontal and temporal regions of FTLD-tau and FTLD-TDP cases, with a much higher burden in white matter (Supplementary Fig. 1d, e). Iba1-positive microglia only differed in the temporal lobe of FTLD-tau cases (Supplementary Fig. 1f).

#### FTLD-tau subtypes

FTLD-Picks cases had a higher burden of all microglial phenotypes compared with controls in the frontal and temporal lobes, except for Iba1-positive microglia in FW, where the burden was low (Fig. 5g–i). FTLD-CBD cases had a higher burden of CD68-positive microglia in the frontal lobe and TW compared with controls, but a similar burden of CR3/43- and Iba1-positive microglia to controls in all regions, except for TW, where the burden of Iba1-positive microglia was higher than controls (Fig. 5g–i). FTLD-PSP and FTLD-*MAPT* cases had a modest burden of Iba1- and CR3/43-positive microglia and a low burden of CD68-positive microglia, similar to controls in most regions (Fig. 5g–i), although in TW FTLD-*MAPT* cases had a higher burden of CD68-positive microglia compared with controls (Fig. 5g).

Comparing cases with underlying tau pathology, FTLD-Picks and FTLD-CBD cases had similar burdens of each microglial phenotype in each region, whereas FTLD-Picks cases had a higher burden of CD68-positive microglia in TW and TG, and CR3/43-positive microglia in all regions, compared with FTLD-PSP cases (Fig. 5g, h). FTLD-Picks cases also had a higher burden of CD68-positive microglia in FW and Iba1-positive microglia in TG compared with FTLD-*MAPT* cases (Fig. 5g, i). FTLD-CBD cases had a higher burden of CD68-positive microglia in the frontal lobe compared with FTLD-*MAPT* cases and in TW compared with FTLD-PSP cases (Fig. 5g) but had a similar burden of CR3/43- and Iba1-positive microglia to other FTLD-tau subtypes (Fig. 5h, i). Grey versus white matter comparisons showed that in most FTLD-tau groups microglial burden was higher in white matter than grey matter, although this varied regionally by FTLD-tau subtype and microglial phenotype (Supplementary Fig. 1g-i).

#### FTLD-TDP subtypes

FTLD-TDPA and FTLD-TDPC cases generally had a high burden of CD68-positive microglia, particularly in white matter, with a significant difference from controls in TW for FTLD-TDPA cases and in FG and FW for FTLD-TDPC cases (Fig. 5j). The burden of CR3/43-positive microglia varied considerably across FTLD-TDPA and FTLD-TDPC cases, particularly in white matter, and overall, there was no significant difference from controls (Fig. 5k). In contrast, FTLD-TDPB cases had a similar burden of CD68- and CR3/43-positive microglia to controls in all regions (Fig. 5j, k). When comparing FTLD-TDP subtypes, the burden of CD68- and CR3/43-positive microglia did not differ between FTLD-TDPA and FTLD-TDPC subtypes in any region but there was a lower burden of burden of CD68- and CR3/43-positive microglia in TG of FTLD-TDPB compared with FTLD-TDPA cases (Fig. 5j, k). The burden of Iba1-positive microglia was similar between FTLD-TDP subtypes and when compared with controls, except for FTLD-TDPC cases, which had a higher burden in FG compared with controls (Fig. 5l). Grey versus white matter comparisons revealed that FTLD-TDPA and FTLD-TDPC cases had a higher burden of CD68- and CR3/43-positive microglia in white than grey matter, particularly in the frontal lobe (Supplementary Fig. 1j, k). FTLD-TDPA cases also had a higher burden of these phenotypes in TW than TG. In contrast, FTLD-TDPB cases had similar burdens of these microglial phenotypes in grey and white matter. The burden of Iba1-positive microglia was similar in white and grey matter within each lobe for all FTLD-TDP subtypes (Supplementary Fig. 1l).

#### Sporadic and genetic FTLD-TDPA subtypes

Sporadic FTLD-TDPA cases had a similar burden of CD68-positive microglia to controls and FTLD-*C9orf72* cases in all regions, but a lower burden of CD68-positive microglia in the temporal lobe compared with FTLD-*GRN* cases (Fig. 5m). FTLD-*GRN* cases had a notably high burden of CD68-positive microglia in the temporal lobe and FW, differing significantly from controls in TW (Fig. 5m). Although many cases, particularly sporadic and FTLD-*GRN* cases, had a high burden of CR3/43-positive microglia in white matter, this varied considerably within each group and did not differ significantly from controls (Fig. 5n). The burden of Iba1-positive microglia was only elevated in the frontal lobe of FTLD-*C9orf72* cases, differing from FTLD-*GRN* cases in FG and FW and controls and sporadic cases in FW (Fig. 5o). Grey versus white matter comparisons revealed that all FTLD-TDPA subtypes had a higher burden of CD68- and CR3/43-positive microglia in white matter than grey matter (Supplementary Fig. 1 m, n). In contrast, the burden of Iba1-positive microglia was similar between white and grey matter for all groups, except for FTLD-*C9orf72* cases, which had a higher burden in FW than FG (Supplementary Fig. 1o).

#### Genetic FTLD subtypes

FTLD-*GRN* cases had a high burden of CD68-positive microglia in most regions, particularly in white matter, but a similar burden to controls in FG (Fig. 5p). FTLD-*C9orf72* cases also had a high burden of CD68-positive microglia in most regions, particularly in white matter (Fig. 5p). In contrast, FTLD-*MAPT* cases only had a higher burden of this phenotype compared with controls in TW, with a lower burden compared with FTLD-*C9orf72* cases in FW. The burden of CR3/43-positive microglia was highly variable across all cases but higher in FW of FTLD-*GRN* cases compared with controls (Fig. 5q). There was a particularly high burden of Iba1-positive microglia in the frontal lobe of FTLD-*C9orf72* cases, differing significantly from controls in FG (Fig. 5r). However, there was a low burden of this phenotype in all regions in FTLD-*GRN* cases, particularly in the frontal lobe (especially FW), where values differed significantly from FTLD-*C9orf72* cases (Fig. 5r). Grey versus white matter comparisons revealed that burdens of CD68- and CR3/43-positive microglia were generally higher in white matter than grey matter in all genetic FTLD subtypes (Supplementary Fig. 1p, q). However, there was no significant difference in the burden of Iba1-positive microglia, apart from a higher burden in FW than FG in FTLD-*C9orf72* cases (Supplementary Fig. 1r).

### Microglial circularity

#### Neurodegenerative disease comparisons

**Fig. 6:**
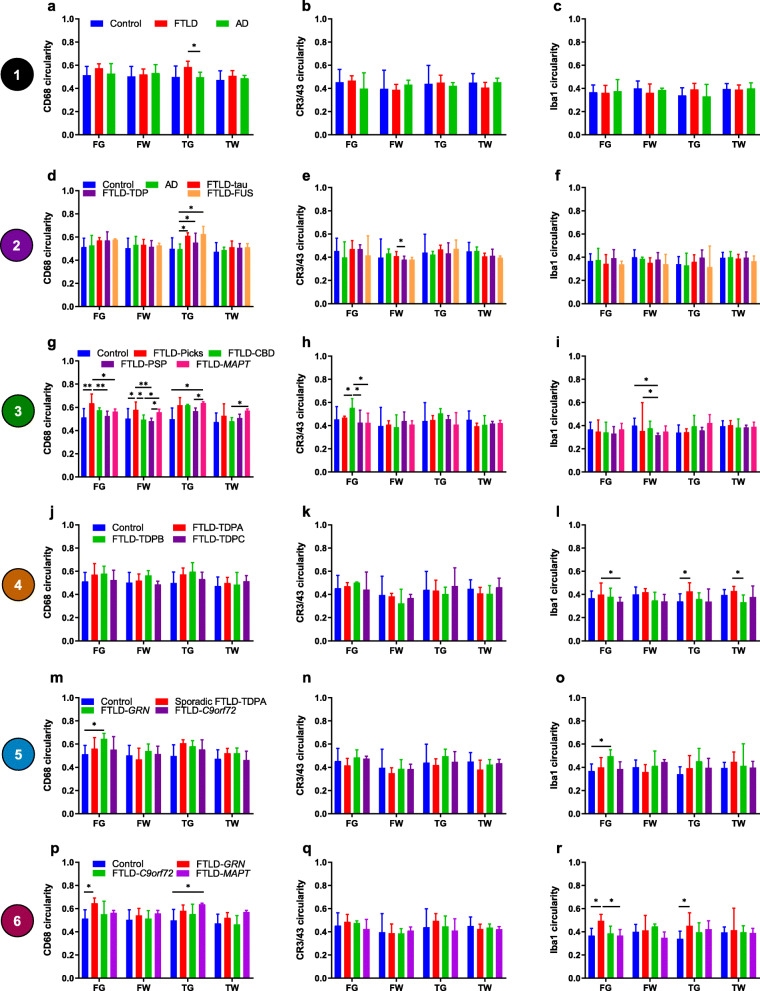
Microglial circularity compared between groups in each region. Comparisons of the circularity of CD68-positive (**a, d, g, j, m, p**), CR3/43-positive (**b, e, h, k, n, q**) and Iba1-positive (**c, f, i, l, o, r**) microglia for each group comparison level shown within Fig. 1 (numbers in coloured circles on the left represent level of comparison). Graphs show median circularity values in each brain region: frontal grey (FG), frontal white (FW), temporal grey (TG) and temporal white (TW) matter. See legend in first graph on each row for bar colours. Error bars represent interquartile range. *p < 0.05; **p < 0.01; ***p ≤ 0.001; ****p ≤ 0.0001

Circularity of CD68-, CR3/43- and Iba1-positive microglia was similar between all groups, apart from FTLD cases having more circular CD68-positive microglia in TG than AD cases (Fig. 6a–c). Grey versus white matter comparisons revealed that FTLD cases had much more circular CD68- and CR3/43-positive microglia in grey matter compared with white matter (Supplementary Fig. 2a, b). There were no differences between grey and white matter for Iba1-positive microglia in any disease group (Supplementary Fig. 2c).

#### Main FTLD subtypes

Circularity of CD68-, CR3/43- and Iba1-positive microglia did not differ between the main FTLD subtypes and controls in any region (Fig. 6d–f) but all three FTLD subtypes had more circular CD68-positive microglia in TG compared with AD cases (Fig. 6d) and FTLD-tau cases had more circular CR3/43-positive microglia in FW compared with FTLD-TDP cases (Fig. 6e). Grey versus white matter comparisons revealed that FTLD-tau and FTLD-TDP cases had more circular CD68- and CR3/43-positive microglia in grey matter than white matter (Supplementary Fig. 2d, e). Circularity of Iba1-positive microglia was similar in grey and white matter for all main FTLD subtypes (Supplementary Fig. 2f).

#### FTLD-tau subtypes

FTLD-Picks cases had more circular CD68-positive microglia in the frontal lobe compared with all other FTLD-tau subtypes and controls (Fig. 6g). Circularity of CD68-positive microglia in FTLD-CBD cases did not differ from controls but was lower in FW compared with FTLD-Picks cases and in FW and TW compared with FTLD-*MAPT* cases (Fig. 6g). FTLD-*MAPT* cases had more circular CD68-positive microglia in TG than controls, in FW and TW compared with FTLD-CBD cases and in FG and TW compared with FTLD-PSP cases (Fig. 6g). Despite a high burden of CR3/43-positive microglia in FTLD-Picks cases in most regions, circularity of this phenotype did not differ significantly from other groups, apart from being lower than FTLD-CBD cases in FG (Fig. 6h). FTLD-CBD cases had very circular CR3/43-positive microglia in FG, differing significantly from all other FTLD-tau groups (Fig. 6h). Circularity of Iba1-positive microglia did not differ between groups, apart from more circular microglia in FW of FTLD-Picks cases and controls compared with FTLD-PSP cases (Fig. 6i). Grey versus white matter comparisons revealed that FTLD-Picks and FTLD-PSP cases had similar circularity values for CD68- and CR3/43-positive microglia in both areas in both lobes (Supplementary Fig. 2g, h). In contrast, FTLD-CBD cases had more circular microglia of both phenotypes in grey matter of both lobes and FTLD-*MAPT* cases had more circular CD68-positive microglia in TG than TW (Supplementary Fig. 2g, h). Circularity of Iba1-positive microglia was similar between areas except for more circular microglia in TW than TG in FTLD-Picks cases (Supplementary Fig. 2i).

#### FTLD-TDP subtypes

There were no significant differences in circularity of CD68- or CR3/43-positive microglia between any FTLD-TDP subtype and controls (Fig. 6j, k). However, FTLD-TDPA cases had more circular Iba1-positive microglia in TG than controls, in TW than FTLD-TDPB cases and in FG compared with FTLD-TDPC cases (Fig. 6l). Grey versus white matter comparisons revealed that FTLD-TDPA cases had more circular CD68- and CR3/43-positive microglia in grey matter than white matter of both lobes (Supplementary Fig. 2j, k), and circularity of CD68-positive microglia in FTLD-TDPC cases was also higher in grey matter of both lobes (Supplementary Fig. 2j), but in FTLD-TDPB cases, circularity values did not differ significantly between grey and white matter. Circularity of Iba1-positive microglia was similar in grey and white matter for each FTLD-TDP subtype (Supplementary Fig. 2l).

#### Sporadic and genetic FTLD-TDPA subtypes

FTLD-*GRN* cases had higher circularity values for CD68- and Iba1-positive microglia in FG compared with controls (Fig. 6m, o). Sporadic FTLD-TDPA and FTLD-*C9orf72* cases had similar circularity values to other groups for all microglial phenotypes in all regions. Grey versus white matter comparisons revealed that circularity values for CD68-positive microglia were generally higher in grey matter than white matter (Supplementary Fig. 2 m). CR3/43-positive microglia were more circular in TG than TW of FTLD-*C9orf72* cases (Supplementary Fig. 2n). Circularity of Iba1-positive microglia was similar between grey and white matter in all groups (Supplementary Fig. 2o).

#### Genetic FTLD subtypes

FTLD-*GRN* cases had more circular CD68-positive microglia than controls in FG and FTLD-*MAPT* cases had more circular CD68-positive microglia than controls in TG (Fig. 6p). However, circularity of this phenotype did not differ between genetic FTLD subtypes in any region. Circularity of CR3/43-positive microglia did not differ between genetic FTLD subtypes or from controls (Fig. 6q). Despite a low burden of Iba1-positive microglia in all regions in FTLD-*GRN* cases, circularity was higher in FG and TG compared with controls, and in FG compared with FTLD-*MAPT* cases (Fig. 6r). Grey versus white matter comparisons revealed that circularity of CD68-positive microglia was generally higher in grey matter than white matter, but this varied regionally according to the mutation: FTLD-*GRN* and FTLD-*C9orf72* cases had more circular CD68-positive microglia in grey matter of both lobes, whereas FTLD-*MAPT* cases only had more circular CD68-positive microglia in TG compared with TW (Supplementary Fig. 2p). FTLD-*C9orf72* cases had more circular CR3/43-positive microglia in FG compared with FW (Supplementary Fig. 2q). Circularity of Iba1-positive microglia did not differ significantly between grey and white matter in any group (Supplementary Fig. 2r).

### Microglial perimeter

#### Neurodegenerative disease comparisons

**Fig. 7:**
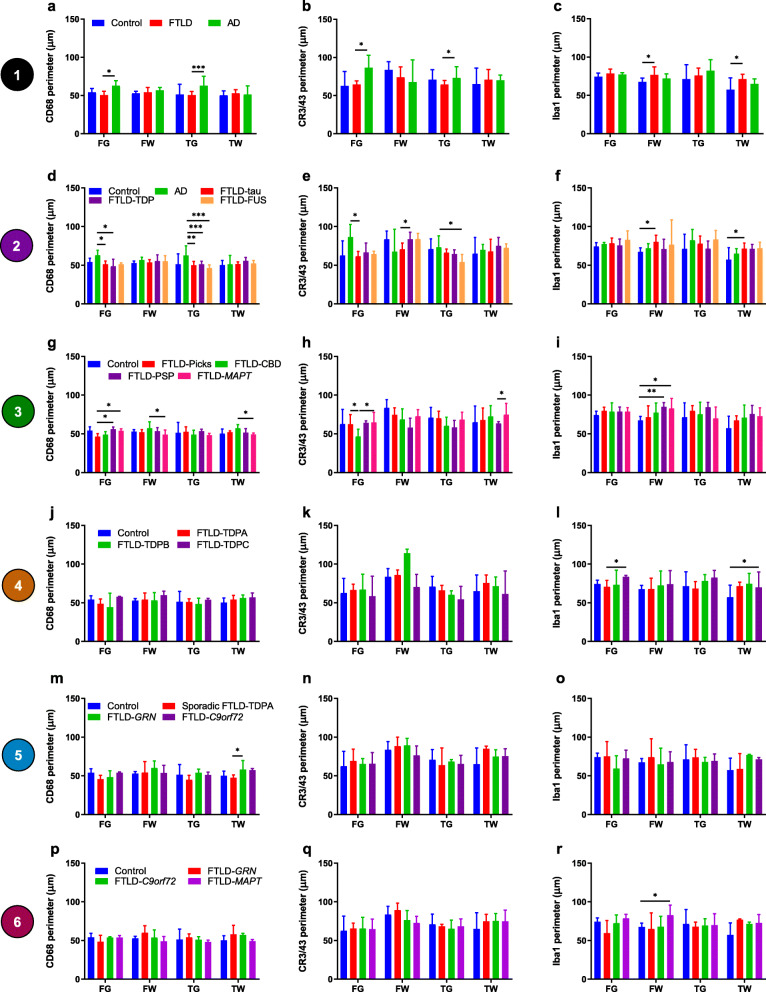
Microglial perimeter compared between groups in each region. Comparisons of the perimeter of CD68-positive (**a, d, g, j, m, p**), CR3/43-positive (**b, e, h, k, n, q**) and Iba1-positive (**c, f, i, l, o, r**) microglia for each group comparison level shown within Fig. 1 (numbers in coloured circles on the left represent level of comparison). Graphs show median perimeter values in each brain region: frontal grey (FG), frontal white (FW), temporal grey (TG) and temporal white (TW) matter. See legend in first graph on each row for bar colours. Error bars represent interquartile range. *p < 0.05; **p < 0.01; ***p ≤ 0.001; ****p ≤ 0.0001

FTLD cases had smaller perimeter CD68- and CR3/43-positive microglia in FG and TG compared with AD cases, but perimeter did not differ significantly from controls (Fig. 7a, b). Iba1-positive microglia had a larger perimeter in FTLD cases in FW and TW compared with controls (Fig. 7c). Grey versus white matter comparisons revealed that FTLD cases had smaller perimeter CD68- and CR3/43-positive microglia in grey matter compared with white matter in both lobes (Supplementary Fig. 3a, b). In contrast, AD cases had smaller perimeter CD68-positive microglia in TW than TG. Controls had smaller perimeter CR3/43-positive microglia in FG than FW. The perimeter of Iba-1 positive microglia was similar in grey and white matter in all groups, except for controls, where it was smaller in TW than TG (Supplementary Fig. 3c).

#### Main FTLD subtypes

All three FTLD subtypes had smaller perimeter CD68-positive microglia in TG compared with AD cases, and perimeter was smaller in FG of FTLD-tau and FTLD-TDP (but not FTLD-FUS) cases compared with AD cases (Fig. 7d). FTLD-tau cases had smaller perimeter CR3/43-positive microglia in FW compared with FTLD-TDP cases, and in FG compared with AD cases, whereas FTLD-FUS cases had smaller perimeter CR3/43-positive microglia in TG compared with AD cases (Fig. 7e). Although circularity of Iba1-positive microglia had been similar across groups, there were larger perimeter Iba1-positive microglia in FW and TW of FTLD-tau cases compared with controls (Fig. 7f). Grey versus white matter comparisons revealed that FTLD-TDP cases had smaller perimeter CD68- and CR3/43-positive microglia in grey matter compared with white matter (Supplementary Fig. 3d, e). FTLD-tau cases also had smaller perimeter CR3/43-positive microglia in grey matter (Supplementary Fig. 3e), but no significant differences for CD68-positive microglia. The perimeter of Iba1-positive microglia did not differ significantly between grey and white matter in any FTLD subtype (Supplementary Fig. 3f).

#### FTLD-tau subtypes

FTLD-Picks cases had smaller perimeter CD68-positive microglia in FG compared with FTLD-PSP and FTLD-*MAPT* cases (Fig. 7g). FTLD-CBD cases had much smaller perimeter CR3/43-positive microglia in FG compared with FTLD-Picks and FTLD-*MAPT* cases, whereas FTLD-PSP cases had smaller perimeter CR3/43-positive microglia in TW than FTLD-*MAPT* cases (Fig. 7h). The perimeter of Iba1-positive microglia was similar across groups, except for FTLD-*MAPT* and FTLD-PSP cases, which had a larger perimeter in FW than controls (Fig. 7i). Grey versus white matter comparisons revealed that FTLD-CBD cases had smaller perimeter CD68- and CR3/43-positive microglia in FG than FW and smaller perimeter CD68-positive microglia in TG than TW (Supplementary Fig. 3 g, h). In contrast, FTLD-Picks cases had smaller perimeter Iba1-positive microglia in TW than TG (Supplementary Fig. 3i). Perimeter did not differ significantly between grey and white matter in FTLD-PSP or FTLD-*MAPT* cases.

#### FTLD-TDP subtypes

Like circularity, the perimeter of CD68- and CR3/43-positive microglia did not differ between FTLD-TDP subtypes and controls in any region (Fig. 7j, k). The perimeter of Iba1-positive microglia was smaller in FG of FTLD-TDPA cases compared with FTLD-TDPC cases but similar in other regions compared with other groups, whereas FTLD-TDPB cases had larger perimeter Iba1-positive microglia than controls in TW (Fig. 7l). Grey versus white matter comparisons revealed smaller perimeter CD68- and CR3/43-positive microglia in grey matter than white matter of both lobes in FTLD-TDPA cases (Supplementary Fig. 3j, k), matching circularity results. In FTLD-TDPC cases, CR3/43-positive microglia were of smaller perimeter in FG than FW (Supplementary Fig. 3k). Perimeter of Iba1-positive microglia was similar in grey and white matter of each FTLD-TDP subtype (Supplementary Fig. 3l).

#### Sporadic and genetic FTLD-TDPA subtypes

The perimeter of CD68-positive microglia was smaller in TW of sporadic FTLD-TDPA cases than FTLD-*GRN* cases (Fig. 7m), despite no difference in circularity between these groups. The perimeter of CR3/43-positive and Iba1-positive microglia did not differ significantly between groups (Fig. 7n, o). Grey versus white matter comparisons showed that perimeter was often smaller in grey matter than white matter. There were smaller perimeter CD68- and CR3/43-positive microglia in FG than FW of sporadic FTLD-TDPA and FTLD-*GRN* cases (Supplementary Fig. 3m, n), and smaller perimeter CD68-positive microglia in TG than TW of FTLD-*GRN* and FTLD-*C9orf72* cases (Supplementary Fig. 3m). The perimeter of Iba1-positive microglia was smaller in TW than TG in sporadic FTLD-TDPA cases (Supplementary Fig. 3o).

#### Genetic FTLD subtypes

Unlike circularity, perimeter did not significantly differ between genetic groups or from controls for any microglial phenotype, apart from larger perimeter Iba1-positive microglia in FW of FTLD-*MAPT* cases compared with controls (Fig. 7p–r). Grey versus white matter comparisons generally revealed smaller perimeter microglia in grey matter than white matter but this varied regionally according to mutation. FTLD-*GRN* cases had smaller perimeter CD68- and CR3/43-positive microglia in grey matter of both lobes (matching circularity results), whereas FTLD-*C9orf72* cases had smaller perimeter of CD68-positive microglia only in TG compared with TW (Supplementary Fig. 3p, q). In FTLD-*MAPT* cases, the perimeter of CD68-positive microglia did not differ significantly between grey and white matter, in contrast to the higher circularity in TG than TW. The perimeter of Iba-1 positive microglia did not differ significantly between grey and white matter in any group (Supplementary Fig. 3r).

### Microglial dystrophy

#### Neurodegenerative disease comparisons

**Fig. 8:**
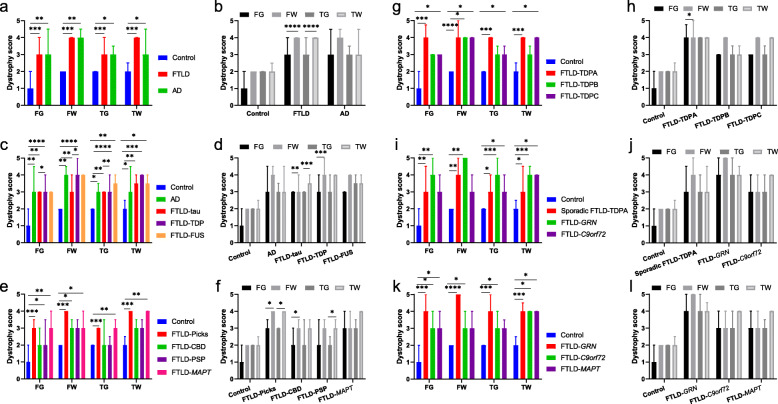
Dystrophy scores compared between groups in each region and between grey and white matter within each lobe for each group. Graphs show comparison of dystrophy scores in each region between groups (**a, c, e, g, i, k**) and between grey and white matter within each lobe for each group (**b, d, f, h, j, l**) for control, FTLD and AD groups (**a, b**), main FTLD subtypes (**c, d**), and subtypes of FTLD-tau (**e, f**), FTLD-TDP (**g, h**), sporadic and genetic FTLD-TDPA (**i, j**) and genetic FTLD (**k, l**). Bars show median dystrophy scores and error bars show interquartile range. FG = frontal grey; FW = frontal white; TG = temporal grey; TW = temporal white matter. *p < 0.05; **p < 0.01; ***p ≤ 0.001; ****p ≤ 0.0001

Dystrophy scores differed significantly between controls, AD and FTLD cases in all regions (Fig. 8a). Controls had no or mild dystrophy in most regions, with most cells appearing intact and ramified. AD cases had more severe dystrophy in all regions compared with controls, with moderate dystrophy in FG and TG, moderate to severe dystrophy in TW, and severe dystrophy in FW. Overall, FTLD cases had much more severe dystrophy than controls in all regions, particularly in white matter, but dystrophy scores were variable across FTLD subtypes. There was no significant difference in the severity of dystrophy between FTLD and AD cases in any region. Grey versus white matter comparisons (Fig. 8b) revealed that dystrophy scores were similar in grey and white matter within each lobe for controls, whereas AD cases had similarly severe dystrophy in FG and FW and a trend towards more severe dystrophy in TW than TG. FTLD cases had more severe dystrophy in white matter than grey matter in both lobes, although this varied across the different FTLD subtypes. Rod-shaped and hypertrophic microglia were infrequent in controls. Rod-shaped microglia were more common in AD and FTLD cases, found mainly in grey matter. Hypertrophic microglia were not seen in controls or AD cases, and in FTLD cases their presence varied by subtype.

#### Main FTLD subtypes

FTLD-tau, FTLD-TDP and FTLD-FUS cases had more severe dystrophy than controls in most regions but dystrophy did not differ significantly between any of these FTLD subtypes and AD cases (Fig. 8c). FTLD-TDP cases had more severe dystrophy than FTLD-tau cases in the frontal lobe and in TG, but not in TW, where dystrophy was particularly severe in many FTLD-tau cases. Grey versus white matter comparisons (Fig. 8d) revealed that dystrophy was more severe in white matter than grey matter for both FTLD-tau and FTLD-TDP cases, but this varied regionally: FTLD-tau cases had much more severe dystrophy in TW than TG but only slightly more severe dystrophy in FW than FG, whereas FTLD-TDP cases had much more severe dystrophy in FW than FG, with a only a trend towards this in TW compared with TG. In contrast, FTLD-FUS cases had similar dystrophy scores in grey and white matter. Rod-shaped and hypertrophic microglia were present in FTLD-tau and FTLD-TDP cases but varied according to pathological subtype. Two of the four FTLD-FUS cases had several rod-shaped microglia in FG. Two other FTLD-FUS cases had no rod-shaped microglia but had several hypertrophic microglia, particularly in frontotemporal white matter.

#### FTLD-tau subtypes

Dystrophy was more severe in most FTLD-tau subtypes compared with controls, but this varied regionally by pathological subtype (Fig. 8e). FTLD-Picks cases had more severe dystrophy than controls in all regions, particularly in white matter, with moderate to severe dystrophy in FG and TG and severe dystrophy in FW and TW. FTLD-CBD cases had mild to moderate dystrophy in FG and TG but moderate to severe dystrophy in FW and TW, differing significantly from controls only in FW. FTLD-PSP cases had mild to moderate dystrophy in all regions, only slightly more severe than controls in FG but not in other regions. Dystrophy was rather variable across FTLD-*MAPT* cases, but overall dystrophy was more severe than controls in all regions. There were no significant differences in dystrophy in any region between the four FTLD-tau subtypes, except for in TW, where FTLD-Picks cases had more severe dystrophy than FTLD-PSP cases. Grey versus white matter comparisons (Fig. 8f) revealed that dystrophy was generally more severe in white than grey matter but again this varied regionally according to pathological subtype. FTLD-Picks cases had more severe dystrophy in FW and TW than FG and TG whereas FTLD-CBD cases had more severe dystrophy in FW than FG only. FTLD-PSP cases had more severe dystrophy in TW than TG only. FTLD-*MAPT* cases had similar dystrophy scores in grey and white matter within both lobes. Rod-shaped microglia were notably scarce in FTLD-Picks cases and FTLD-PSP cases. In contrast, all FTLD-CBD cases and four out of five FTLD-*MAPT* cases had several rod-shaped microglia in FG and TG, and all FTLD-*MAPT* cases had several rod-shaped microglia in FW or TW. Most FTLD-Picks cases had numerous hypertrophic microglia in FG and TG, but these were typically not visible in FW or TW, where there was more severe dystrophy. Hypertrophic cells were infrequent or absent in other FTLD-tau subtypes.

#### FTLD-TDP subtypes

Dystrophy was more severe in most FTLD-TDP subtypes in most regions compared with controls, but this varied by pathological subtype (Fig. 8g). FTLD-TDPA cases had more severe dystrophy than controls in all regions, but dystrophy was particularly severe in FW of FTLD-*GRN* cases, and the FTLD-TDPA case with a *TBK1* mutation, which had severe to very severe dystrophy in all regions. Dystrophy in FTLD-TDPB cases differed from controls only in FW and was more variable between cases. The sporadic FTLD-TDPB cases (both of whom had FTD-MND) had moderate to severe dystrophy in all regions, worse in white matter, and of the three genetic FTLD-TDPB cases with *C9orf72* mutations, one with behavioural variant frontotemporal dementia (bvFTD) had mild dystrophy in three out of four regions but moderate dystrophy in TW, and the other two cases (bvFTD or FTD-MND) had moderate dystrophy in three regions but severe dystrophy in the remaining region (FW in bvFTD and TG in FTD-MND). FTLD-TDPC cases had more homogeneous dystrophy scores, with more severe dystrophy than controls in all regions. Although many FTLD-TDPA and FTLD-TDPC cases appeared to have more severe dystrophy in most regions than FTLD-TDPB cases, this was variable across cases and did not reach significance at a group level in any region. Grey versus white matter comparisons (Fig. 8h) showed that dystrophy was generally more severe in FW than FG, but similar in TW and TG, in all FTLD-TDP subtypes. This difference was most noticeable for FTLD-TDPA cases, who had tended to have severe or very severe dystrophy in FW. The presence of rod-shaped microglia varied according to pathological subtype: several FTLD-TDPA cases had rod-shaped microglia, mainly those with *C9orf72* expansions or sporadic cases. Occasional rod-shaped microglia were present in grey matter of sporadic FTLD-TDPB cases, but they were frequent in FTLD-TDPB cases with *C9orf72* expansions. One FTLD-TDPC case had rod-shaped microglia in FG, but these were infrequent. All FTLD-TDPC cases had frequent, prominently hypertrophic microglia in frontotemporal grey and white matter (particularly white matter), whereas these were not seen frequently in FTLD-TDPA or FTLD-TDPB cases.

#### Sporadic and genetic FTLD-TDPA subtypes

Dystrophy was more severe in sporadic FTLD-TDPA and FTLD-*GRN* cases than controls in all regions, whereas FTLD-*C9orf72* cases had more severe dystrophy than controls only in the temporal lobe (Fig. 8i). There was a trend towards more severe dystrophy in FW of FTLD-*GRN* cases compared with FTLD-*C9orf72* cases. Grey versus white matter comparisons (Fig. 8j) revealed that all subtypes generally had similarly severe dystrophy scores in grey and white matter, although there was a trend towards more severe dystrophy in FW than FG of FTLD-*GRN* cases. Occasional rod-shaped microglia were seen in FG of sporadic FTLD-TDPA cases, but these were frequent in FTLD-*C9orf72* cases, particularly in FG. Rod-shaped microglia were not visible in any FTLD-*GRN* cases due to extensive loss of cell structure. Hypertrophic microglia were not observed in any group.

#### Genetic FTLD subtypes

**Fig. 9:**
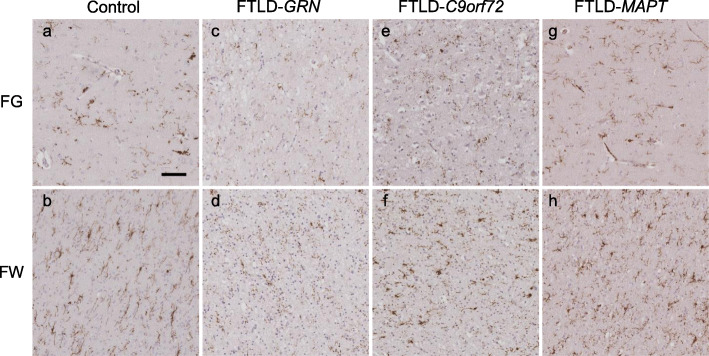
Examples of dystrophic microglia in controls and genetic FTLD subtypes. Differing severities of dystrophic Iba1-positive microglia are visible in frontal grey (FG) matter (**a, c, e, g**) and frontal subcortical white (FW) matter (**b, d, f, h**) of controls (**a, b**) and FTLD-*GRN* (**c, d**), FTLD-*C9orf72* (**e, f**) and FTLD-*MAPT* (**g, h**) cases. Scale bar represents 50 μm in all images. Note that in the FTLD-*GRN* case there is particularly extensive dystrophy, which is worse in white matter, where there is generalised punctate Iba1 staining consistent with severe cellular disruption (**d**). Images were taken from the following cases (in Supplementary Table 1): control (case 4), FTLD-*GRN* (case 40, has FTLD-TDPA), FTLD-*C9orf72* (case 42, has FTLD-TDPA) and FTLD-*MAPT* (case 16)

Dystrophy was rather variable across cases within genetic FTLD groups, except for FTLD-*GRN* cases, who all had severe to very severe dystrophy in all regions, differing significantly from controls in all regions (Fig. 8k), especially in FW (Fig. 9d). Although in this analysis FTLD-*C9orf72* cases had more severe dystrophy than controls in all regions, dystrophy appeared less consistently severe on an individual case basis than for FTLD-*GRN* cases. There was a trend towards more severe dystrophy in FW of FTLD-*GRN* cases compared with FTLD-*C9orf72* cases, despite both groups having FTLD-TDPA (Fig. 9d, f). However, out of the FTLD-*C9orf72* group, three had FTD-MND; one had mild dystrophy in FG and TG but moderate dystrophy in FW and TW, the second had severe dystrophy throughout the frontal lobe but moderate dystrophy in the temporal lobe, and the third had moderate dystrophy in FG but severe dystrophy in all other regions. The remaining two FTLD-*C9orf72* cases had nfvPPA; one had severe dystrophy in all regions except FW (moderate) and in the other dystrophy was mild in FG, moderate in FW and TG and severe in TW. FTLD-*MAPT* cases generally had moderate to severe dystrophy in most regions, with more severe dystrophy than controls in FG (but not in FW; Fig. 8k and Fig. 9h) and much more severe dystrophy in TW. Despite most FTLD-*MAPT* cases appearing to have less severe dystrophy than FTLD-*GRN* or FTLD-*C9orf72* cases, at the group level this did not reach significance in any region. Grey versus white matter comparisons revealed that FTLD-*GRN* cases had a trend towards more severe dystrophy in FW than FG (Fig. 8l and Fig. 9c, d), but in other groups dystrophy was similarly severe in grey and white matter.

Frequent rod-shaped microglia were present in frontotemporal grey matter of *C9orf72* expansion carriers with FTLD-TDPA. Although not included in the FTLD-*C9orf72* group for the semi-quantitative dystrophy analysis, all three FTLD-TDPB cases with *C9orf72* expansions also had numerous rod-shaped microglia in grey matter. Four out of five FTLD-*MAPT* cases had several rod-shaped microglia in grey matter and white matter. Rod-shaped microglia were not visible in any FTLD-*GRN* cases due to extensive dystrophy. Hypertrophic microglia were not observed in any genetic FTLD cases.

## Discussion

This study performed a comprehensive, quantitative assessment of the burden and activation state of three different microglial phenotypes and the severity of microglial dystrophy in frontotemporal grey and white matter in post-mortem brain tissue from a large and diverse cohort of individuals with sporadic and genetic FTLD, sporadic AD and healthy controls. This approach has demonstrated that microglia differ between individuals with a neurodegenerative disease and healthy controls, and between clinically overlapping neurodegenerative diseases (FTLD and AD). Regional patterns of microglial burden, activation and dystrophy differ across the spectrum of neuropathology within FTLD, not only between the main FTLD subtypes (FTLD-tau, FTLD-TDP and FTLD-FUS), but also between different pathological subtypes of FTLD-tau and FTLD-TDP, between individuals with the same pathology due to different causes (sporadic and genetic FTLD-TDPA), and between individuals with genetic FTLD due to different mutations (FTLD-*GRN*, *FTLD-C9orf72* and FTLD-*MAPT*). Importantly, microglial burden and activation also varied according to the microglial phenotype analysed. Microglial burden, activation and dystrophy varied between grey and white matter in FTLD, with generally more microglia present in white matter, but these are in a less activated and more dystrophic state. Qualitative assessment of rod-shaped and hypertrophic microglia has also demonstrated that these unusual cell morphologies are present in FTLD, but that their presence varies by pathological subtype.

The low burden and limited activation of microglia in controls was unsurprising, due to a relatively young median group AAD (similar to the overall FTLD group), and has been shown in previous studies [10, 39]. The high burden of phagocytic microglia in frontotemporal grey and white matter of FTLD cases is consistent with a previous study of this microglial phenotype in an FTLD cohort [39] and FTLD-TDP cases [44]. However, the use of three different microglial markers in the present study has enabled appreciation that overall, FTLD is associated with a higher burden of phagocytic (CD68-positive) microglia in frontotemporal regions compared with controls, without a concurrent increase in antigen-presenting (CR3/43-positive) microglia. Although many FTLD cases had a low burden of Iba1-positive microglia, this was later shown to be due to extensive dystrophy and consequently reduced cell staining in most regions. We found more phagocytic microglia in FG of AD cases compared with controls and FTLD cases, differing from a previous study [39]. However, cohort composition and analysis techniques differed from our study.

Although there was a high burden of phagocytic ‘looking’ microglia in FTLD and AD, analysis of morphological parameters of activation has demonstrated that they were not well activated by the end stage of disease. This contradicts other studies showing greater activation of this phenotype in frontotemporal regions of FTLD and AD cases compared with controls [39, 44]. In FTLD cases, the burden of phagocytic and antigen-presenting microglia was higher in frontotemporal white matter than grey matter, but activation was greater in grey matter. This indicates that there is a microglial ‘white matter signal’ in FTLD, with an influx of microglial phenotypes with key immune functions into white matter. These results support early descriptions of white matter microgliosis in FTLD [33–35, 37, 38] and recent studies showing more phagocytic microglia in white matter than grey matter of FTLD cases [39, 44]. Neuroimaging studies of FTD demonstrate early, widespread loss of white matter integrity, preceding grey matter atrophy, which varies regionally by clinical and genetic phenotype [49, 56, 57], occurs presymptomatically [48, 58] and progresses over time [58, 59]. This could be a primary process, occurring prior to protein aggregation, or a secondary process, in response to retrograde axonal degeneration from neuronal loss due to cortical pathology.

Findings in all FTLD cases were replicated in FTLD-tau and FTLD-TDP, where the burden of phagocytic microglia was similar in FTLD-tau and FTLD-TDP cases, as identified previously [39]. However, FTLD-tau cases had more Iba1-positive microglia in frontotemporal white matter compared with FTLD-TDP and AD cases, and in TW compared with controls, perhaps due to less severe dystrophy, or more extensive white matter tau pathology. The FTLD-FUS group had a low burden of all microglial phenotypes, with limited activation, in all regions. ALS mouse models expressing wild-type FUS display a pro-inflammatory microglial phenotype and excessive release of inflammatory cytokines [60]. However, all FTLD-FUS cases were previously diagnosed as aFTLD-U, which tend to have more severe pathology in the hippocampus and subcortical grey and brainstem nuclei, with less cortical and subcortical white matter involvement [46]. Grey versus white matter comparisons of microglia in FTLD-tau and FTLD-TDP also showed that the burden of phagocytic and antigen-presenting microglia was higher in white matter than grey matter, but these phenotypes were more activated in grey matter, suggesting that microglial dysfunction as well as activation occurs in regions with extensive pathology. However, burden and activation were similarly low in grey and white matter of FTLD-FUS cases. Given that there is extensive white matter pathology in both FTLD-tau and FTLD-TDP [61], there may be selectively impaired activation of certain microglial phenotypes in white matter. There have been few previous comparisons of microglia between grey and white matter in FTLD-tau and FTLD-TDP, and none in FTLD-FUS. In other studies, FTLD-tau [39] and FTLD-TDP [39, 43, 44] cases had more phagocytic microglia in frontotemporal white matter compared with grey matter, but microglia were also more activated in white matter.

A previous study did not distinguish fully between the different FTLD-tau subtypes [39]. However, we found that FTLD-Picks cases had numerous phagocytic and antigen-presenting microglia in frontotemporal regions, and particularly significant activation of phagocytic microglia in white matter, consistent with the frontal and white matter predominance of pathology found in this subtype [46, 62]. Extensive microglial burden and activation have previously been described in FTLD-Picks [23, 33, 63, 64]. FTLD-CBD cases had intermediate levels of microgliosis but significant activation of phagocytic and antigen-presenting microglia in the frontal lobe, particularly grey matter, reflecting the regional pathological distribution of tau pathology [46]. A microglial PET study identified extensive binding suggestive of increased microglial activation in CBD [65], but in the present study, the lack of microglial activation in most regions in FTLD-CBD cases suggests there may be dysfunction of this phenotype in areas of particularly high tau burden by the end stage of disease. FTLD-PSP cases had a low burden and limited activation of all microglial phenotypes in frontotemporal regions, but other studies of FTLD-PSP have shown pronounced microgliosis in subcortical and infratentorial structures, correlating with tau burden [35, 66], and that activation detected using microglial PET occurs in subcortical but not cortical regions [67]. FTLD-*MAPT* cases had a moderate burden of microglia in most regions, but numerous phagocytic microglia in the temporal lobe, particularly in white matter, consistent with a previous study [39], although in our study microglia were more activated in grey matter than white matter. These different microglial patterns may be due to, or contribute to, regional differences in tau pathology, especially different tau strains or cell-specific tau burdens. A recent study using the microglial PET radioligand ^11^C-PK-11195 showed that binding correlates regionally with binding of a radioligand (^18^F-AV-1451) that localises to pathology [23]. The relationship between microglia and regional burdens of different tau pathologies could be explored using radioligands more specific for various tau strains, once available.

FTLD-TDPA cases had prominent temporal microgliosis, whilst FTLD-TDPC cases had prominent frontal microgliosis. Both FTLD-TDPA and FTLD-TDPC were associated with more phagocytic and antigen-presenting microglia in white matter, but greater activation in grey matter. Although FTLD-TDPC cases typically have more pronounced temporal lobe pathology than FTLD-TDPA cases, both subtypes have significant frontal pathology by the end stage of disease [46]. FTLD-TDPB cases had a low burden of all microglial phenotypes in all regions; most had FTD-MND, which may have been associated with less temporal pathology than the range of clinical presentations in FTLD-TDPA cases. Other studies also found numerous phagocytic microglia in frontotemporal grey and white matter of FTLD-TDPA and FTLD-TDPB cases [39, 44]. More phagocytic microglia were present in white matter than grey matter in other studies of FTLD-TDPA and FTLD-TDPC cases, but due to different analysis methods, activation was also deemed greater in white matter [39, 43].

Analysis of FTLD-TDPA subtypes revealed that different causes of TDP-43 pathology are associated with different microglial patterns in each region. Sporadic FTLD-TDPA was associated with an abundance of poorly activated antigen-presenting microglia in frontotemporal white matter, whereas FTLD-*GRN* was associated with an abundance of phagocytic microglia in the temporal lobe, but these were only well activated in FG. FTLD-*C9orf72* was associated with a high burden of Iba1-positive microglia, particularly in the frontal lobe. Mouse models of *GRN* mutations and *C9orf72* expansions clearly demonstrate microglial activation and dysfunction, but mechanisms in sporadic FTLD-TDPA remain unclear. This variation in a range of microglial phenotypes within cortical regions across FTLD-TDPA subtypes suggests that different underlying disease mechanisms influence microglia in individuals with the same pathology.

We showed that different genetic FTLD subtypes are associated with different regional and phenotypic patterns of microglia. However, again there was a higher burden in white matter but greater activation in grey matter, suggesting microglia are dysfunctional in white matter in genetic FTLD. Results in the FTLD-*GRN* group support findings in a single FTLD-*GRN* case, containing many phagocytic and antigen-presenting microglia in frontotemporal grey and white matter, but few Iba1-positive microglia in FW [54], although others have found many phagocytic and Iba1-positive microglia in the frontal cortex of FTLD-*GRN* cases [53, 68]. Although many antigen-presenting microglia were present in FW, these were not well activated. Dysfunctional presentation and targeting of self-antigens in white matter (such as myelin) could contribute to the prominent, early, white matter damage seen in FW of *GRN* mutation carriers, including demyelination and white matter hyperintensities [54, 57, 58]. FTLD-*C9orf72* cases had abundant phagocytic microglia within frontotemporal white matter, and many Iba1-positive microglia but few antigen-presenting microglia in all regions. Extensive frontal pathology in the FTLD-*C9orf72* group could underlie this, or this could be a mutation-dependent effect, given that antigen-presenting microglia were not increased. Mouse models of the *C9orf72* expansion display numerous phagocytic or Iba1-positive microglia with enhanced activation in a region-specific manner prior to neuronal loss and TDP-43 aggregation [69]. However, we found that all microglial phenotypes were poorly activated in FTLD-*C9orf72* cases. Microglia in FTLD-*MAPT* cases were only altered in the temporal lobe, with an increased burden of phagocytic microglia in TW, but greater activation in TG, compared with controls. This is consistent with regional tau pathology patterns and phagocytic microglia in *MAPT* 10 + 16 mutation carriers [39, 46]. *MAPT* mouse models and microglial PET studies suggest that regionally selective microglial activation and dysfunction occurs several years before other neuroimaging changes and symptom onset, preceding tau pathology [26, 70–72]. Our study confirms this remains the case by the end stage of disease.

This study also assessed the severity of microglial dystrophy across the spectrum of FTLD and AD. Dystrophy was minimal in controls and similarly severe in FTLD and AD cases. However, the severity of dystrophy in FTLD varied regionally according to underlying pathology, disease mechanism and gene mutation. Severe dystrophy has been demonstrated in AD [13, 15, 16, 19, 21, 22, 55, 73], but most studies examined hippocampal regions. Microglial dystrophy has not been compared previously between FTLD and AD, but as dystrophy was similarly severe in all regions, this suggests that there is a common mechanism of excessive microglial senescence. Few studies have examined dystrophy in FTLD, mainly in genetic cases, but these indicate that dystrophy varies regionally and according to potential disease mechanism [53, 54], and our results support this. More severe dystrophy was generally observed in white matter than grey matter and this may explain the poor activation of phagocytic and antigen-presenting microglia, and low burden of Iba1-positive microglia, in white matter of most FTLD and AD cases. If microglia are more vulnerable to dysfunction or senescence in white matter, this may predispose individuals to onset of pathology in white matter due to lack of axonal support, or influence spread of pathology through white matter tracts. FTLD cases with *GRN* mutations show early, region-specific white matter neuroimaging abnormalities on MRI, such as white matter hyperintensities (WMH), which are not due to vascular disease and occur presymptomatically [54, 58]. As microglial function varies regionally with age [14], the regional differences in dystrophy observed across pathological and genetic FTLD subtypes suggest that regional variations in senescence may determine focal onset of certain neurodegenerative pathologies.

This study extends the few previous studies of microglial dystrophy in genetic FTLD, which have focused on FTLD-*GRN* or FTLD-*C9orf72* [42, 53, 54] and describes dystrophy in FTLD-*MAPT* for the first time. Dystrophy was consistently extensive, particularly in FW, in all regions in FTLD-*GRN* cases, where there was punctate, diffuse Iba1 staining consistent with total loss of cell integrity and generalised distribution of microglial debris [16]. This replicates findings from a single FTLD-*GRN* case where dystrophy was extensive in white matter and correlated regionally with MRI WMH and white matter pathology [54]. Recently, lipid droplet accumulating microglia have been implicated in the pathogenesis of FTLD due to *GRN* mutations. This could play a role in microglial dysfunction and early senescence. Although previously identified in aging [74] and AD [18], microglia in *GRN* knockout mouse models display severe accumulation of lipids, significant defects in phagocytosis, increased reactive oxygen species and elevated pro-inflammatory cytokines [74]. This extends previous research showing microglial lipofuscin accumulation [75] and foam cell formation in GRN-deficient macrophages [76] and accumulation of triacylglycerides in fibroblast and lysosomal lipidomes in humans and mice with *GRN* mutations [77]. Dystrophy was more severe in FTLD-*C9orf72* cases in all regions compared with controls but varied considerably across cases within this group. Multiple studies indicate that *C9orf72* expansions cause microglial dysfunction through impaired lysosomal mechanisms (reviewed in [78]), but senescence may vary regionally, perhaps modified by variants in lysosomal genes such as *TMEM106B* [79], which could contribute to the heterogeneity of clinical phenotypes in expansion carriers. Dystrophy in FTLD-*MAPT* cases was more severe than controls in FG and TW, which matches regional burdens of tau pathology [39, 46], but dystrophy was also rather variable across cases. Many dystrophic, senescent microglia are present in *MAPT* mouse models and these promote tau hyperphosphorylation and aggregation, neurodegeneration and cognitive dysfunction [72, 80]. Clearance of senescent cells prevents these changes [80], so anti-senescence therapies may be a promising approach for further exploration in models of FTD.

Microglial dystrophy is clearly altered in both sporadic and genetic FTLD, so lipid handling, lysosomal function or other pathways contributing to senescence could be valuable therapeutic targets. However, microglial function is complex and is likely influenced not only by mutations in FTD-associated genes known to affect microglial or lysosomal function (such as *GRN*, *C9orf72*, *MAPT*, *TBK1*, *SQSTM1*, *VCP* or *CHMP2B*), but also by polygenic variants in immune system genes linked to FTD, such as *HLA* loci [81, 82] or *TREM2* [83, 84] and in lysosomal genes linked to variability in FTLD-*GRN* and FTLD-*C9orf72* such as *TMEM106B*, *SORT1* and *PSAP* [79, 85–88]. Variants in immune pathway or lysosomal genes linked to variability in sporadic disease, such as FTLD-TDP (*HLA-DQA2*, *DHX58*, *TRIM21*, *IRF7*) [89], and multiple other external and environmental factors, may play a role in determining the fate of microglia in an aging individual that develops sporadic disease.

Rod-shaped microglia were infrequent in controls, but frequently observed in AD and FTLD cases, although this varied by FTLD subtype and were most common in grey matter of FTLD-TDP cases with *C9orf72* expansions, FTLD-CBD cases and FTLD-*MAPT* cases. These cells are highly motile and thought to be an intermediate state between ramified and amoeboid morphology [90]. Rod-shaped microglia have been described in AD cases co-located with Aβ and tau pathology [13, 55], but have not previously been reported in frontotemporal regions. Previous studies have examined rod-shaped microglia in FTLD-TDPA, identifying regional differences linked to different disease mechanisms [42] or gene mutations [53], but rod-shaped microglia have not previously been examined across the wider spectrum of disease. Several FTLD cases, mainly those with FTLD-TDPC or FTLD-Picks, had very hypertrophic microglia in frontotemporal grey matter, suggestive of primed, activated microglia [13], but these were not present in AD cases. The role of rod-shaped and hypertrophic microglia remains unclear, but variability in microglial morphology across the spectrum of FTLD may be due to different pathologies or gene mutations impacting on microglial function. Rod-shaped microglia were frequent in grey matter of FTLD-TDPA with *C9orf72* expansions, as described previously [53] and in FTLD-TDPB cases with *C9orf72* expansions. Rod-shaped microglia have not been described in FTLD-*MAPT* cases previously but seem to be prominent in both grey and white matter. Although rod-shaped microglia were not visible in FTLD-*GRN* cases, most likely due to extensive dystrophy, others have found many rod-shaped microglia in hippocampal CA1 regions [42] or the middle frontal cortex [53] of FTLD-*GRN* cases. How specific mutations are linked to the formation of rod-shaped microglia, and how regional variations in these cells link to regional neurodegeneration patterns in genetic FTLD, remains unclear. Hypertrophic microglia were not observed in any genetic group, supporting quantitative findings of limited microglial activation in most genetic FTLD cases, although these may have been difficult to identify in most genetic FTLD cases due to the degree of dystrophy.

Limitations of this study included the relatively small subgroup sizes and small number of brain regions analysed, which may have limited appreciation of the full extent of microglial changes across the spectrum of disease, and our power to detect subtle differences in microglia between subgroups or sub-regions. However, small group sizes (particularly for rarer pathologies such as FTLD-FUS or sporadic FTLD-TDPB) are inherent to human post-mortem studies of a disease as diverse and rare as FTLD. Hence, we decided to analyse a wide selection of cases with different pathologies, balanced with a reasonable number of cases in each subgroup, to allow meaningful FTLD subtype comparisons. Controls were selected to have a similar group-level AAD to the FTLD and AD groups overall, but one control was only 38 years old at the time of death, so all controls and cases were not strictly ‘age-matched’. Younger individuals have less microglial activation than older individuals, and this may have affected microglial comparisons between certain groups, but there were few controls available in our centre who had a similar AAD to the (relatively young) median AAD of the FTLD group.

It is difficult to prove through histological assessments of human post-mortem brain tissue that the morphological appearance of a microglial cell represents a particular activation state or function, how that relates to nearby pathology, or whether or when this plays a role in disease. We are examining the end stage of the disease process, so there may be different phases of microglial involvement, which change over time. There could be excessive microglial activation in association with evolving pathology much earlier on in the disease process, including many years presymptomatically, followed by eventual microglial senescence. In addition, variability in staining quality between cases in histological studies can affect reliability of quantitative assessments, including those based on percentage area stained (used here to quantify microglial burden) and cell morphology (used to quantify activation). We used narrow hue and saturation thresholds during analysis of DAB staining to limit the effects of this, but this cannot always fully adjust for significant variability. It is also not possible to confirm using histological techniques such as immunohistochemistry whether different markers expressed by microglia truly indicate the predominant function or dysfunction of cells detected within a region, particularly as one cell is likely to express a variety of markers at one time, which may change rapidly over time. How expression of these markers relates to dysfunction of microglia or other glial cells, neurons or axons and formation of specific pathological aggregates, also remains unclear.

## Conclusions

In conclusion, our study assessed the burden and activation state of a range of different microglial phenotypes and quantified the severity of microglial dystrophy across a wide variety of sporadic and genetic FTLD subtypes, compared with AD cases and controls. This has allowed appreciation that distinct phenotypes of microglia are altered in each region and that this varies regionally according to FTLD subtype, disease mechanism and gene mutation, and between grey and white matter. Although there are often many phagocytic and antigen-presenting microglia present in FTLD cases, these are not well activated, and microglia are often severely dystrophic, particularly in white matter, suggestive of increased senescence and extensive microglial dysfunction, which may contribute to regional vulnerability to neurodegeneration. Future correlation of these findings with regional microglial PET imaging changes in presymptomatic and symptomatic individuals with different clinical phenotypes or gene mutations could aid differentiation of pathology based on regional microglial patterns in vivo*.* Correlation of histopathological changes in microglia with regional neuropathology patterns in a larger cohort and analysis of a wider range of brain regions would explore our hypothesis that regional changes in microglia reflect regional burdens and types of pathology. Use of induced pluripotent stem cell or mouse models to study the effects of different protein aggregates and gene mutations on microglial activation, function and senescence should aid understanding of the timeline, extent and mechanisms of microglial dysfunction in FTLD. This may guide whether immunomodulatory or anti-senescence therapies could be candidates for future clinical trials in individuals with presymptomatic or symptomatic FTD.

## Supplementary information

**Additional file 1: Supplementary Table 1.** Demographics and diagnoses of all individual cases and controls. **Supplementary Fig. 1.** Microglial burden compared between grey and white matter within each lobe for each group. Comparisons of the burden of CD68-positive (a, d, g, j, m, p), CR3/43-positive (b, e, h, k, n, q), and Iba1-positive (c, f, i, l, o, r) microglia for each group comparison level shown within Fig. 1 (numbers in coloured circles on left represent level of comparison). Graphs show median microglial burden (percentage area values) compared within lobes: frontal grey (FG) versus frontal white (FW) matter, and temporal grey (TG) versus temporal white (TW) matter. See legend in first graph on each row for bar colours. Error bars represent interquartile range. *p < 0.05; **p < 0.01; ***p ≤ 0.001; ****p ≤ 0.0001. **Supplementary Fig. 2.** Microglial circularity compared between grey and white matter within each lobe for each group. Comparisons of the circularity of CD68-positive (a, d, g, j, m, p), CR3/43-positive (b, e, h, k, n, q), and Iba1-positive (c, f, i, l, o, r) microglia for each group comparison level shown within Fig. 1 (numbers in coloured circles on left represent level of comparison). Graphs show median circularity values compared within lobes: frontal grey (FG) versus frontal white (FW) matter, and temporal grey (TG) versus temporal white (TW) matter. See legend in first graph on each row for bar colours. Error bars represent interquartile range. *p < 0.05; **p < 0.01; ***p ≤ 0.001; ****p ≤ 0.0001. **Supplementary Fig. 3.** Microglial perimeter compared between grey and white matter within each lobe for each group. Comparisons of the perimeter of CD68-positive (a, d, g, j, m, p), CR3/43-positive (b, e, h, k, n, q), and Iba1-positive (c, f, i, l, o, r) microglia for each group comparison level shown within Fig. 1 (numbers in coloured circles on left represent level of comparison). Graphs show median perimeter values compared within lobes: frontal grey (FG) versus frontal white (FW) matter, and temporal grey (TG) versus temporal white (TW) matter. See legend in first graph on each row for bar colours. Error bars represent interquartile range. *p < 0.05; **p < 0.01; ***p ≤ 0.001; ****p ≤ 0.0001

**Additional file 2: Supplementary Fig. 4.** Heat map of comparisons of microglial burden between all groups and between grey and white matter within each group. a and b: CD68-positive microglia; c and d: CR3/43-positive microglia; e and f: Iba1-positive microglia. Frontal lobe: a, c, e. Temporal lobe: b, d, f. *P* values are presented in each box and represent results of comparisons between groups listed on corresponding vertical versus horizontal axes. The colour of each box represents the degree of statistical significance in the difference between groups, with red indicating a highly significant difference, blue a non-significant difference and white borderline (trend) or moderately significant difference, with gradations in between. FG = frontal grey matter; FW = frontal white matter; TG = temporal grey matter; TW = temporal white matter. **Supplementary Fig. 5.** Heat map of comparisons of microglial circularity between all groups and between grey and white matter within each group. a and b: CD68-positive microglia; c and d: CR3/43-positive microglia; e and f: Iba1-positive microglia. Frontal lobe: a, c, e. Temporal lobe: b, d, f. *P* values are presented in each box and represent results of comparisons between groups listed on corresponding vertical versus horizontal axes. The colour of each box represents the degree of statistical significance in the difference between groups, with red indicating a highly significant difference, blue a non-significant difference and white borderline (trend) or moderately significant difference, with gradations in between. FG = frontal grey matter; FW = frontal white matter; TG = temporal grey matter; TW = temporal white matter. **Supplementary Fig. 6.** Heat map of comparisons of microglial perimeter between all groups and between grey and white matter within each group. a and b: CD68-positive microglia; c and d: CR3/43-positive microglia; e and f: Iba1-positive microglia. Frontal lobe: a, c, e. Temporal lobe: b, d, f. *P* values are presented in each box and represent results of comparisons between groups listed on corresponding vertical versus horizontal axes. The colour of each box represents the degree of statistical significance in the difference between groups, with red indicating a highly significant difference, blue a non-significant difference and white borderline (trend) or moderately significant difference, with gradations in between. FG = frontal grey matter; FW = frontal white matter; TG = temporal grey matter; TW = temporal white matter. **Supplementary Fig. 7.** Heat map of comparisons of microglial dystrophy scores between all groups and between grey and white matter within each group. a frontal lobe; b temporal lobe. *P* values are presented in each box and represent results of comparisons between groups listed on corresponding vertical versus horizontal axes. The colour of each box represents the degree of statistical significance in the difference between groups, with red indicating a highly significant difference, blue a non-significant difference and white borderline (trend) or moderately significant difference, with gradations in between. FG = frontal grey matter; FW = frontal white matter; TG = temporal grey matter; TW = temporal white matter.

## Data Availability

The datasets used and/or analysed during the current study are available from the corresponding author on reasonable request.

## Abbreviations

AD: Alzheimer’s disease
AAD: Age at death
AAO: At onset of symptoms
aFTLD-U: Atypical frontotemporal lobar degeneration with ubiquitinated inclusions
bvFTD: Behavioural variant frontotemporal dementia
CBD: Corticobasal degeneration
CBS: Corticobasal syndrome
CD68: Cluster of differentiation 68
*C9orf72*: Chromosome 9 open reading frame 72 (gene)
DAB: 3,3′Di-aminobenzidine
FG: Frontal grey matter
FTD: Frontotemporal dementia
FTLD: Frontotemporal lobar degeneration
FW: Frontal white matter
FUS: Fused in sarcoma
*GRN*: Progranulin (gene)
HLA-DP/DQ/DR: Human leucocyte antigen related protein P/Q/R
Iba1: Ionised calcium-binding adaptor molecule 1
lvPPA: Logopenic variant primary progressive aphasia
*MAPT*: Microtubule-associated protein tau (gene)
MHC: Major histocompatibility complex
nfvPPA: Nonfluent variant primary progressive aphasia
PPA: Primary progressive aphasia
PSP: Progressive supranuclear palsy
PSPS: Progressive supranuclear palsy syndrome
svPPA: Semantic variant primary progressive aphasia
TG: Temporal grey matter
TW: Temporal white matter
TDP: TDP-43
*TBK1*: TANK-binding kinase 1 (gene)
WMH: White matter hyperintensities
