# Supplementary material for: Microglial burden, activation and dystrophy patterns in frontotemporal lobar degeneration
Source: J Neuroinflammation. 2020 Aug 10;17:234. doi: 10.1186/s12974-020-01907-0 (PMC7418403; doi:10.1186/s12974-020-01907-0)

Additional File 1: Supplementary Table 1 and Supplementary Figures 1 to 3

Supplementary Table 1. Demographics of all cases and controls. na, not available.

Case code	Pathological diagnosis	Clinical diagnosis	Genetic mutation	Sex	Age at onset (years)	Age at death (years)	Disease duration (years)	Post-mortem delay (hours)	Brain weight (grams)
**Controls**									
1	Normal / pathological aging	Control	-	F	-	80	-	49.2	1242
2	Normal	Control	-	F	-	68	-	45.1	1330
3	Normal	Control	-	M	-	38	-	80.6	1581
4	Normal / pathological aging	Control	-	F	-	73	-	24.0	1214
5	Normal / pathological aging	Control	-	F	-	78	-	29.5	1225
**AD**									
6	AD	AD	-	F	65	70	5	46.9	1233
7	AD	AD	-	M	65	72	7	38.9	1325
8	AD	AD	-	M	52	69	17	35.1	891
9	AD	AD	-	M	63	73	10	31.2	1269
10	AD	AD	-	F	49	62	13	76.7	996
**FTLD-tau**									
11	FTLD-CBD	nfvPPA	-	F	65	73.8	8.8	37.3	996
12	FTLD-CBD	PSPS	-	F	64	69.5	5.5	80.8	1100
13	FTLD-CBD	nfvPPA	-	M	57	68.0	11.0	81.6	980
14	FTLD-CBD	CBS	-	F	58	69.1	11.1	103.3	917
15	FTLD-CBD	nfvPPA	-	M	57	64.8	7.8	41.4	1137
16	FTLD-*MAPT*	bvFTD	*MAPT 10+16*	M	58	66.4	8.4	58.2	1399
17	FTLD-*MAPT*	bvFTD	*MAPT 10+16*	M	37	52.8	15.8	52.6	1046
18	FTLD-*MAPT*	bvFTD	*MAPT 10+16*	F	52	68.4	16.4	24.0	na
19	FTLD-*MAPT*	bvFTD	*MAPT 10+16*	F	43	52.4	9.4	64.0	na
20	FTLD-*MAPT*	bvFTD	*MAPT 10+16*	F	50	58.1	8.1	31.0	na
21	FTLD-Picks	bvFTD	-	M	64	75.6	11.6	46.5	933
22	FTLD-Picks	bvFTD	-	M	58	63.5	5.5	24.0	1166
23	FTLD-Picks	bvFTD	-	M	55	65.6	10.6	43.5	1040
24	FTLD-Picks	bvFTD	-	M	52	67.6	15.6	30.5	982
25	FTLD-Picks	bvFTD	-	M	59	68.1	9.1	94.8	1209
26	FTLD-PSP	PSPS	-	F	65	74.0	9.0	72.5	1182
27	FTLD-PSP	PSPS	-	M	62	68.0	6.0	25.6	na
28	FTLD-PSP	PSPS	-	M	70	78.3	8.3	86.4	1249
29	FTLD-PSP	PSPS	-	M	67	73.7	6.7	69.3	1303
30	FTLD-PSP	nfvPPA	-	M	71	84.0	13.0	32.6	1137
**FTLD-TDP**									
31	FTLD-TDPA	FTD-MND	-	F	76	78.6	2.6	36.3	1119
32	FTLD-TDPA	bvFTD	-	M	63	75.2	10.2	41.0	na
33	FTLD-TDPA	nfvPPA	-	F	65	64.4	2.4	na	na
34	FTLD-TDPA	FTD-MND	-	M	62	53.1	2.1	54.0	na
35	FTLD-TDPA	bvFTD	-	M	51	62.0	4.0	92.9	na
36	FTLD-TDPA	bvFTD	*TBK1 A705fs*	M	58	72.2	9.2	97.4	1320

Case code	Pathological diagnosis	Clinical diagnosis	Genetic mutation	Sex	Age at onset (years)	Age at death (years)	Disease duration (years)	Post-mortem delay (hours)	Brain weight (grams)
37	FTLD-TDPA	bvFTD	*GRN Q130fs*	F	62	68.1	6.1	99.8	na
38	FTLD-TDPA	bvFTD	*GRN C31fs*	M	53	61.4	8.4	72.6	994
39	FTLD-TDPA	nfvPPA	*GRN C31fs*	F	67	74.2	7.2	157.6	1025
40	FTLD-TDPA	bvFTD	*GRN C31fs*	F	58	63.8	5.8	85.4	851
41	FTLD-TDPA	bvFTD	*GRN C31fs*	M	50	55.3	5.3	29.4	974
42	FTLD-TDPA	FTD-MND	*C9orf72*	F	68	75.1	7.1	85.8	782
43	FTLD-TDPA	nfvPPA	*C9orf72*	F	57	67.3	10.3	85.6	789
44	FTLD-TDPA	nfvPPA	*C9orf72*	F	55	62.7	7.7	63.1	981
45	FTLD-TDPA	FTD-MND	*C9orf72*	M	66	71.7	5.7	51.9	1431
46	FTLD-TDPA	FTD-MND	*C9orf72*	F	58	66.3	8.3	107.1	850
47	FTLD-TDPB	bvFTD	*C9orf72*	F	60	66.0	6.0	94.1	1186
48	FTLD-TDPB	bvFTD	*C9orf72*	M	54	62.1	8.1	na	na
49	FTLD-TDPB	FTD-MND	*C9orf72*	F	54	59.0	5.0	na	na
50	FTLD-TDPB	FTD-MND	-	F	63	67.2	4.2	45.5	1232
51	FTLD-TDPB	FTD-MND	-	M	50	56.2	6.2	10.8	na
52	FTLD-TDPC	svPPA	-	F	59	73.0	14.0	37.9	976
53	FTLD-TDPC	svPPA	-	F	55	73.7	18.7	83.7	936
54	FTLD-TDPC	svPPA	-	M	64	78.6	14.6	26.8	1110
55	FTLD-TDPC	svPPA	-	M	64	74.3	10.3	19.0	1230
56	FTLD-TDPC	svPPA	-	M	52	65.4	13.4	51.8	1057
**FTLD-FUS**									
57	FTLD-FUS (aFTLD-U)	bvFTD	-	M	44	51.4	7.4	24.0	na
58	FTLD-FUS (aFTLD-U)	bvFTD	-	M	51	60.4	9.4	48.0	na
59	FTLD-FUS (aFTLD-U)	bvFTD	-	M	47	52.5	5.5	72.0	na
60	FTLD-FUS (aFTLD-U)	bvFTD	-	M	40	51.3	11.3	12.0	na

Supplementary Fig.1 Microglial burden compared between grey and white matter within each lobe for each group. Comparisons of the burden of CD68-positive (a, d, g, j, m, p), CR3/43-positive (b, e, h, k, n, q), and Iba1-positive (c, f, i, l, o, r) microglia for each group comparison level shown within Fig. 1 (numbers in coloured circles on left represent level of comparison). Graphs show median microglial burden (percentage area values) compared within lobes: frontal grey (FG) versus frontal white (FW) matter, and temporal grey (TG) versus temporal white (TW) matter. See legend in first graph on each row for bar colours. Error bars represent interquartile range. *p<0.05; ** p<0.01; *** p≤0.001; ****p≤0.0001.

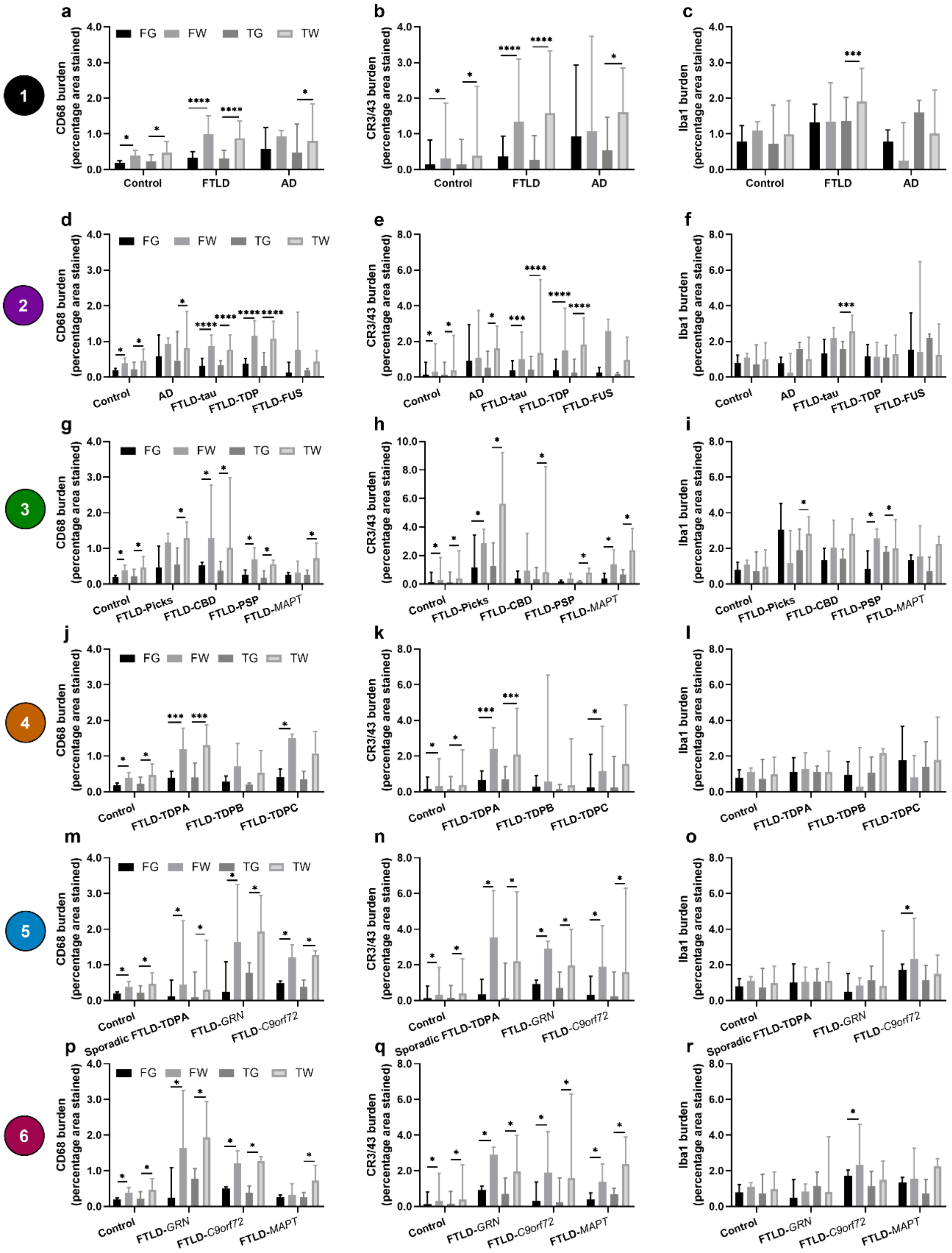

Woollacott *et al.* Microglial burden, activation and dystrophy patterns in frontotemporal lobar degeneration

Supplementary Fig. 2 Microglial circularity compared between grey and white matter within each lobe for each group. Comparisons of the circularity of CD68-positive (a, d, g, j, m, p), CR3/43-positive (b, e, h, k, n, q), and Iba1-positive (c, f, i, l, o, r) microglia for each group comparison level shown within Fig. 1 (numbers in coloured circles on left represent level of comparison). Graphs show median circularity values compared within lobes: frontal grey (FG) versus frontal white (FW) matter, and temporal grey (TG) versus temporal white (TW) matter. See legend in first graph on each row for bar colours. Error bars represent interquartile range. **p*<0.05; ** *p*<0.01; *** *p*≤0.001; **** *p*≤0.0001

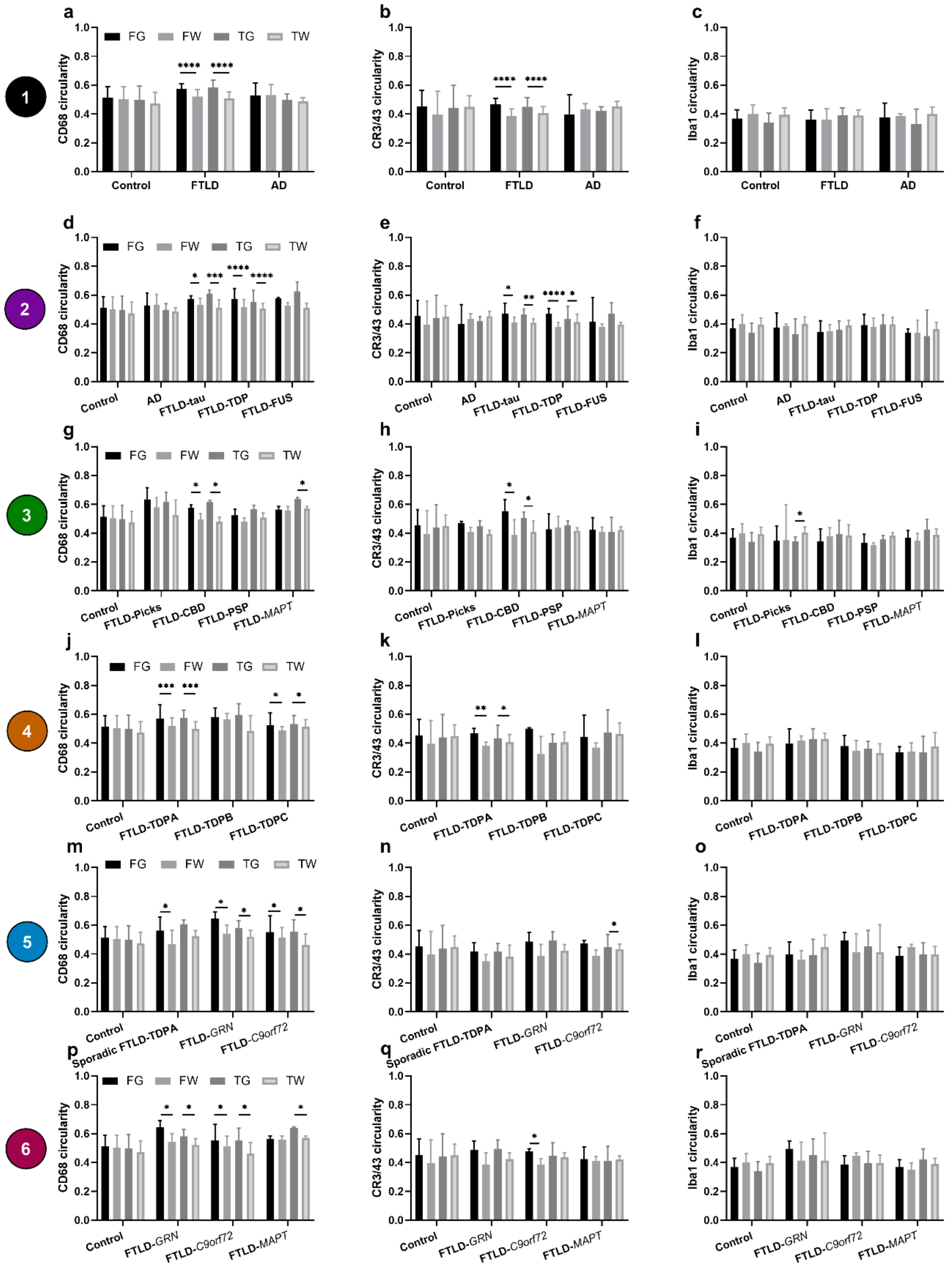

Supplementary Fig. 3 Microglial perimeter compared between grey and white matter within each lobe for each group. Comparisons of the perimeter of CD68-positive (a, d, g, j, m, p), CR3/43-positive (b, e, h, k, n, q), and Iba1-positive (c, f, i, l, o, r) microglia for each group comparison level shown within Fig. 1 (numbers in coloured circles on left represent level of comparison). Graphs show median perimeter values compared within lobes: frontal grey (FG) versus frontal white (FW) matter, and temporal grey (TG) versus temporal white (TW) matter. See legend in first graph on each row for bar colours. Error bars represent interquartile range. *p<0.05; ** p<0.01; *** p≤0.001; **** p≤0.0001.

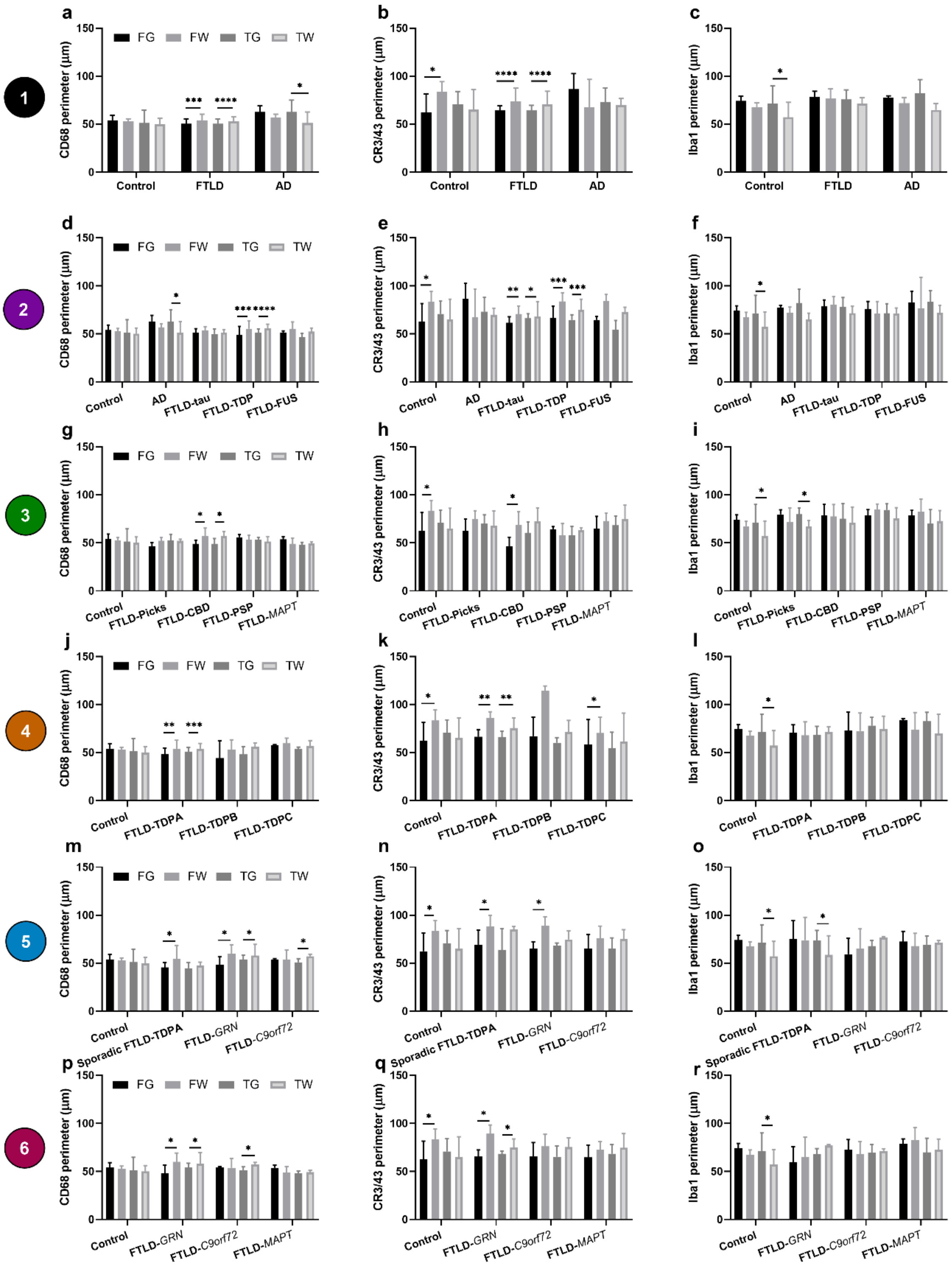

Supplement: Supplementary file 1 — Additional file 1: Supplementary Table 1. Demographics and diagnoses of all individual cases and controls. Supplementary Fig. 1. Microglial burden compared between grey and white matter within each lobe for each group. Comparisons of the burden of CD68-positive (a, d, g, j, m, p), CR3/43-positive (b, e, h, k, n, q), and Iba1-positive (c, f, i, l, o, r) microglia for each group comparison level shown within Fig. 1 (numbers in coloured circles on left represent level of comparison). Graphs show median microglial burden (percentage area values) compared within lobes: frontal grey (FG) versus frontal white (FW) matter, and temporal grey (TG) versus temporal white (TW) matter. See legend in first graph on each row for bar colours. Error bars represent interquartile range. *p < 0.05; **p < 0.01; ***p ≤ 0.001; ****p ≤ 0.0001. Supplementary Fig. 2. Microglial circularity compared between grey and white matter within each lobe for each group. Comparisons of the circularity of CD68-positive (a, d, g, j, m, p), CR3/43-positive (b, e, h, k, n, q), and Iba1-positive (c, f, i, l, o, r) microglia for each group comparison level shown within Fig. 1 (numbers in coloured circles on left represent level of comparison). Graphs show median circularity values compared within lobes: frontal grey (FG) versus frontal white (FW) matter, and temporal grey (TG) versus temporal white (TW) matter. See legend in first graph on each row for bar colours. Error bars represent interquartile range. *p < 0.05; **p < 0.01; ***p ≤ 0.001; ****p ≤ 0.0001. Supplementary Fig. 3. Microglial perimeter compared between grey and white matter within each lobe for each group. Comparisons of the perimeter of CD68-positive (a, d, g, j, m, p), CR3/43-positive (b, e, h, k, n, q), and Iba1-positive (c, f, i, l, o, r) microglia for each group comparison level shown within Fig. 1 (numbers in coloured circles on left represent level of comparison). Graphs show median perimeter values compared within lobes: frontal grey (F [file 12974_2020_1907_MOESM1_ESM.pdf]
