# Supplementary material for: Microglial burden, activation and dystrophy patterns in frontotemporal lobar degeneration
Source: J Neuroinflammation. 2020 Aug 10;17:234. doi: 10.1186/s12974-020-01907-0 (PMC7418403; doi:10.1186/s12974-020-01907-0)

Additional File 2: Supplementary Figures 4 to 7

Supplementary Fig. 4 Heat map of comparisons of microglial burden between all groups and between grey and white matter within each group. a and **b:** CD68-positive microglia; **c** and **d:** CR3/43-positive microglia; **e** and **f**: Iba1-positive microglia. Frontal lobe: **a, c, e**. Temporal lobe: **b, d, f**. *P* values are presented in each box and represent results of comparisons between groups listed on corresponding vertical versus horizontal axes. The colour of each box represents the degree of statistical significance in the difference between groups, with red indicating a highly significant difference, blue a non-significant difference and white borderline (trend) or moderately significant difference, with gradations in between. FG = frontal grey matter; FW = frontal white matter; TG = temporal grey matter; TW = temporal white matter

a

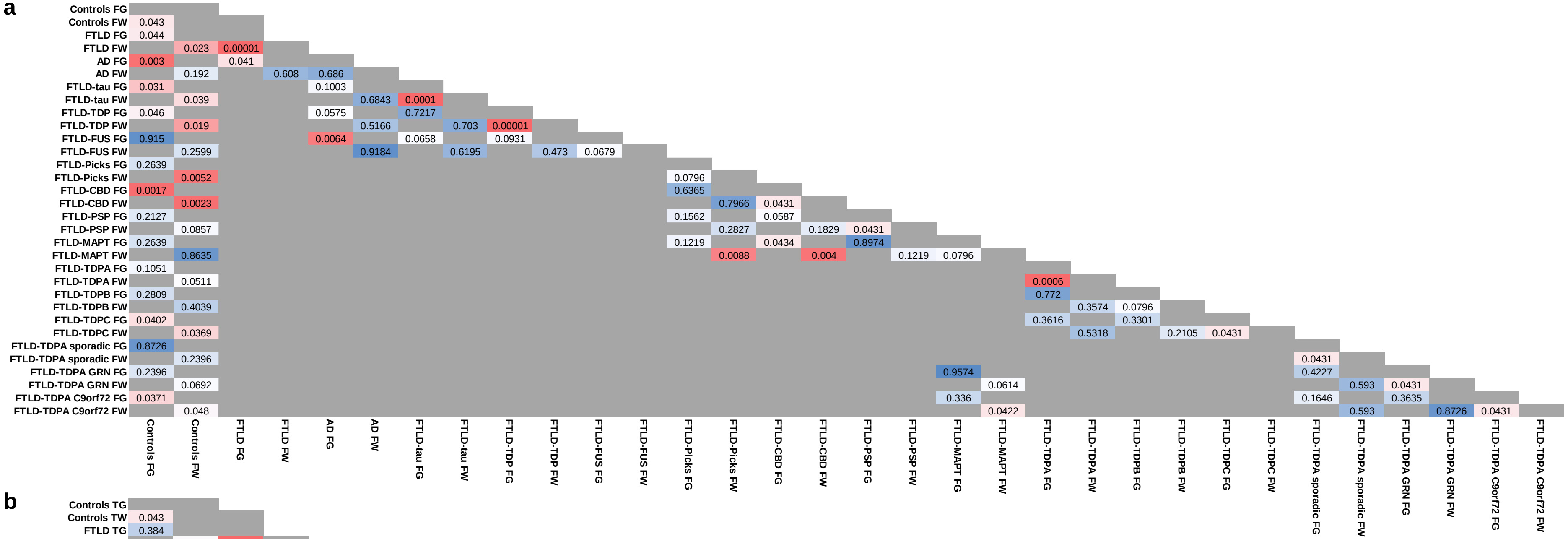

b

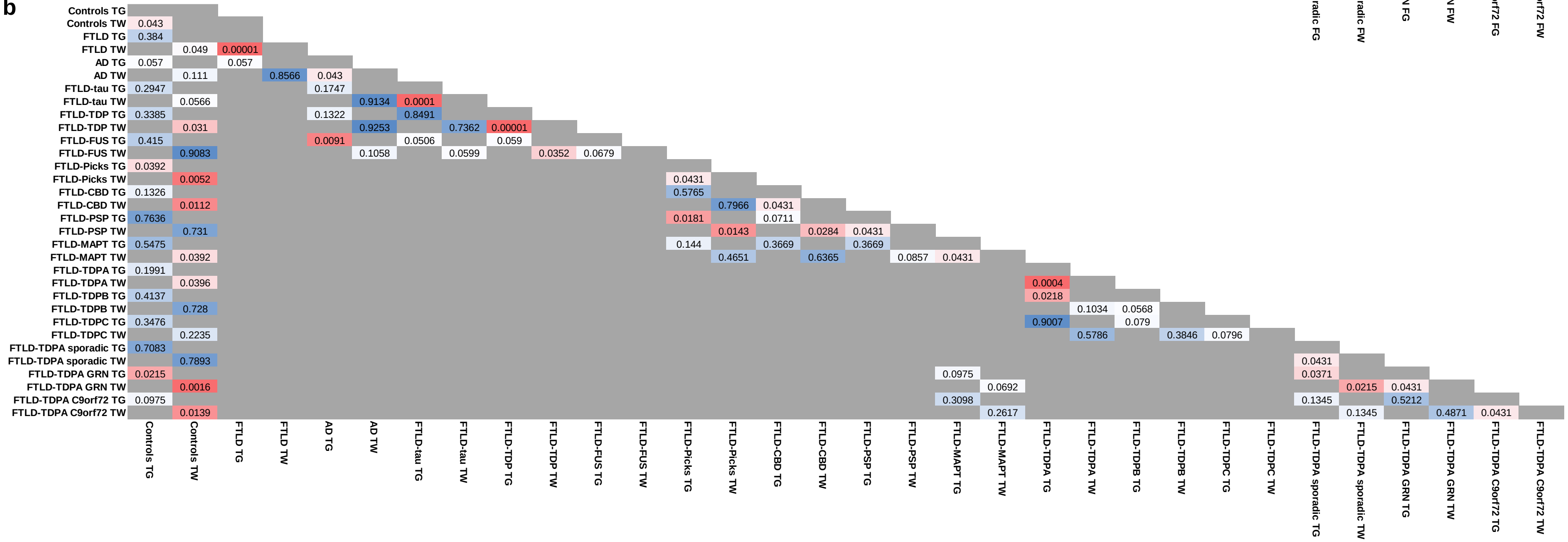

Supplementary Fig. 4

c

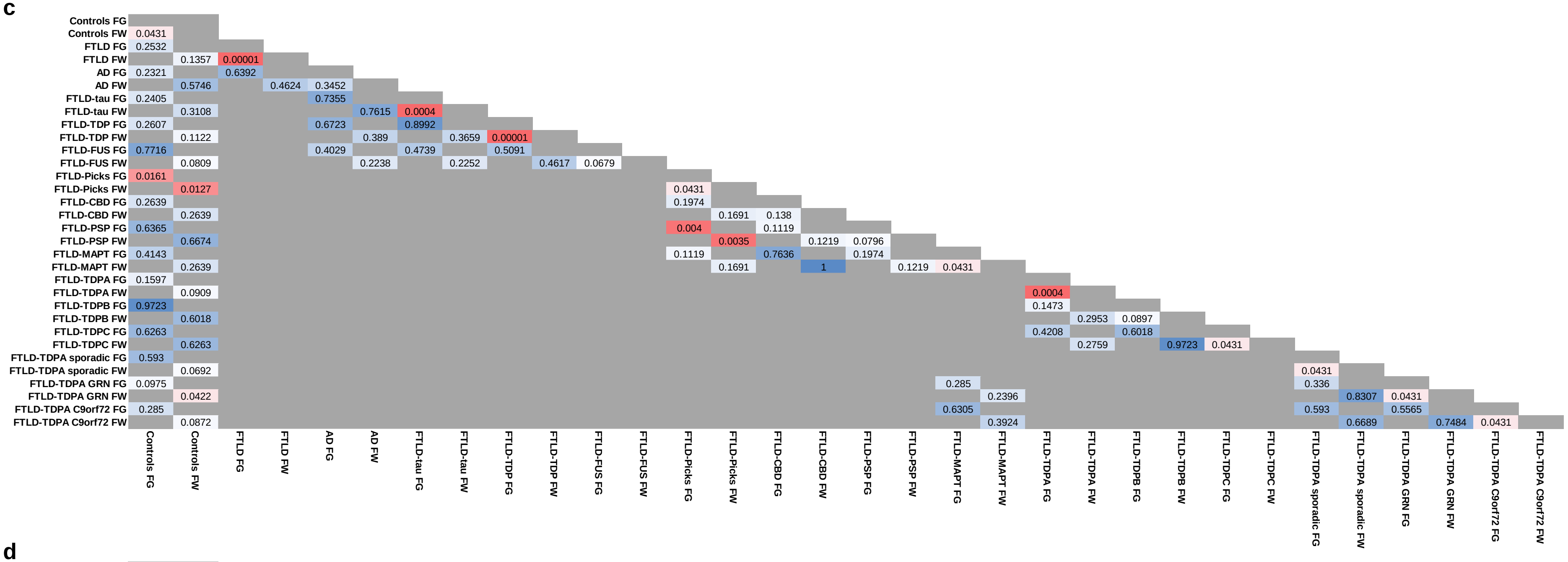

d

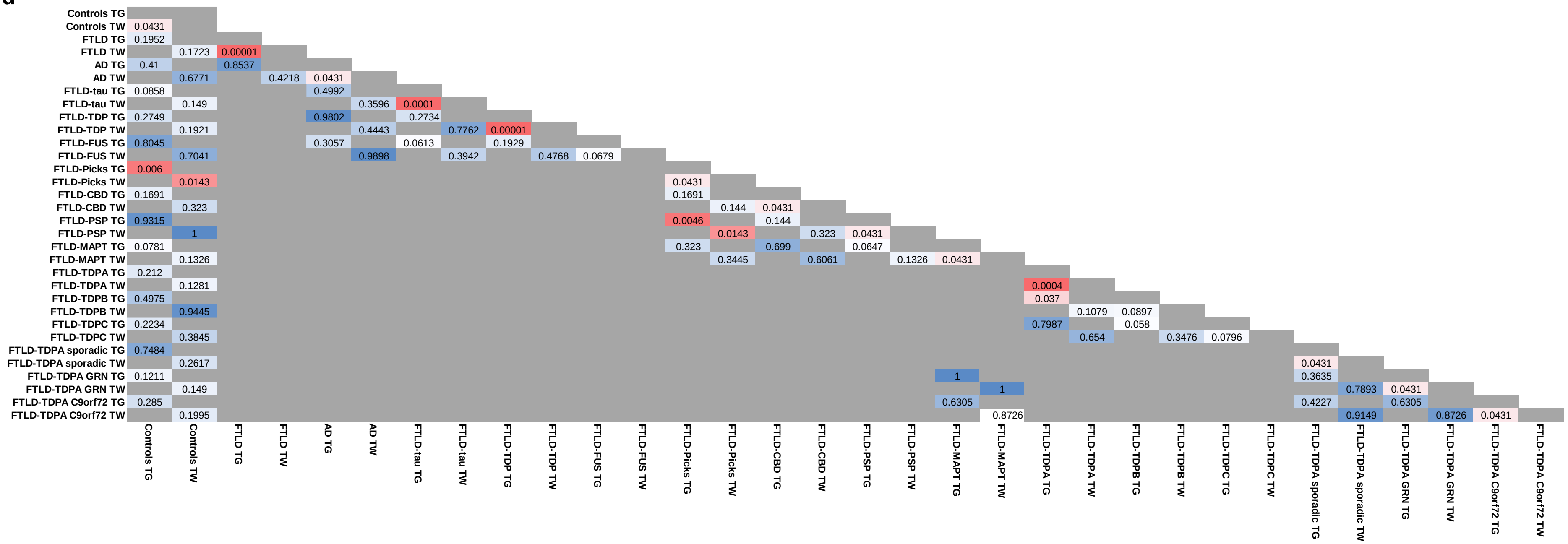

Supplementary Fig. 4

e

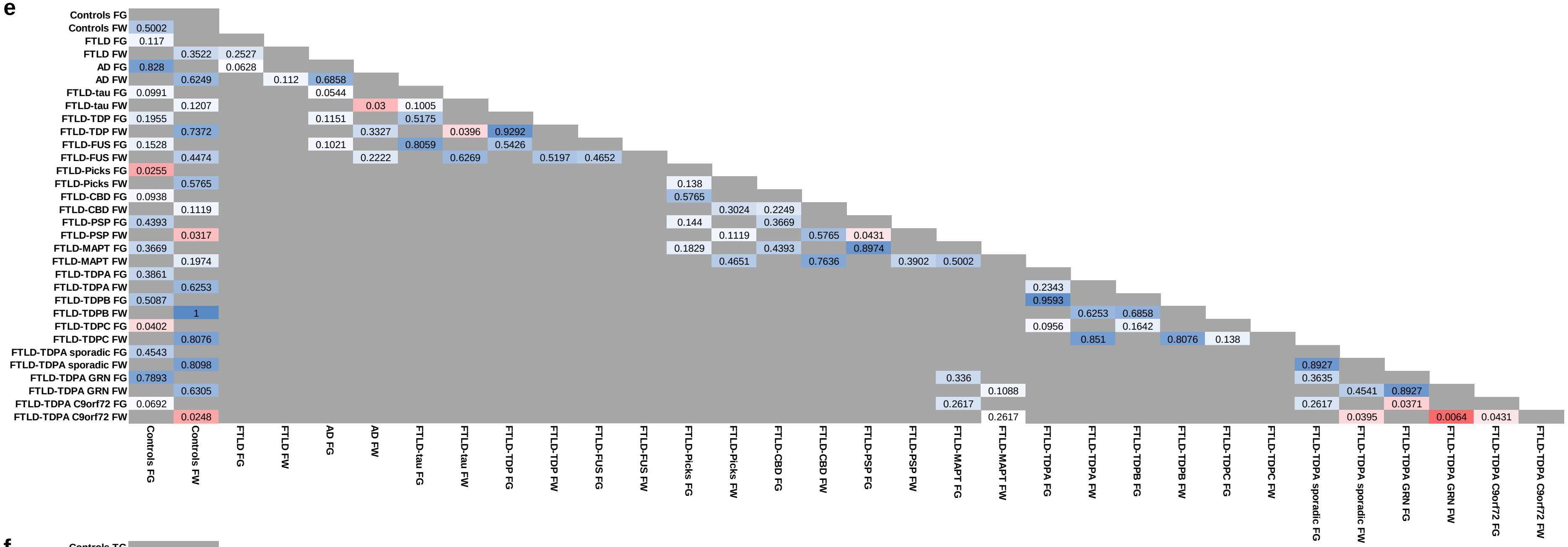

f

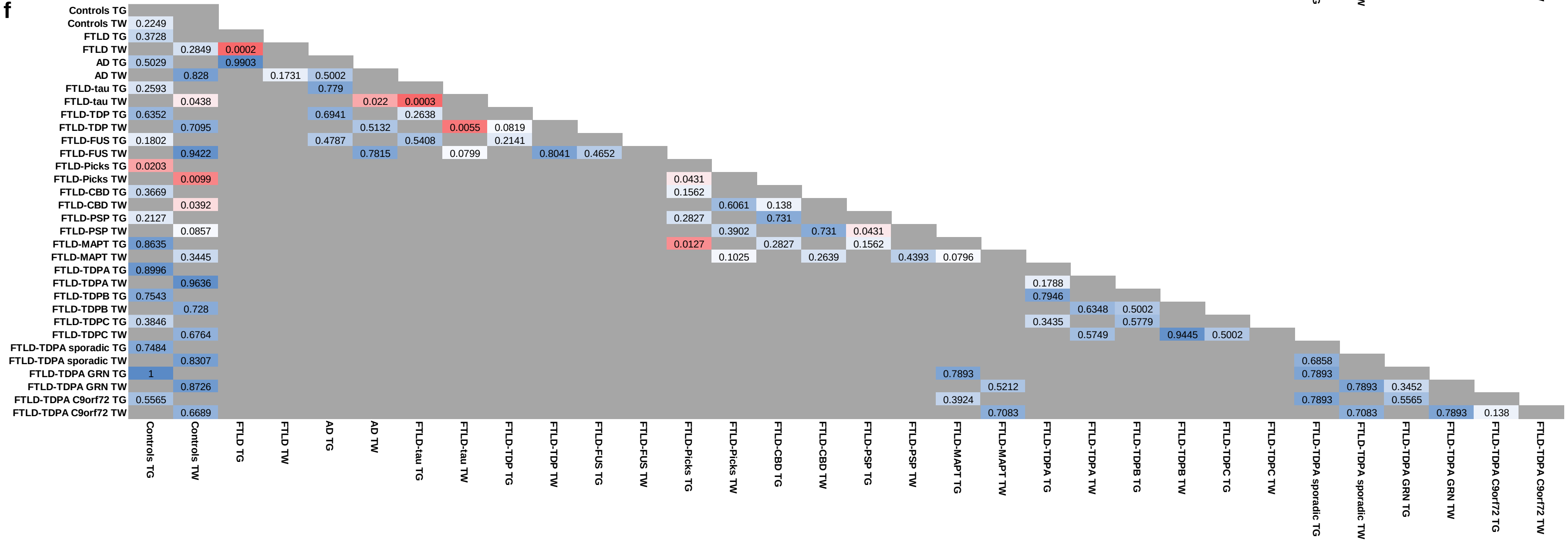

Supplementary Fig. 5 Heat map of comparisons of microglial circularity between all groups and between grey and white matter within each group. a and **b:** CD68-positive microglia; **c** and **d:** CR3/43-positive microglia; **e** and **f:** Iba1-positive microglia. Frontal lobe: **a, c, e**. Temporal lobe: **b, d, f.** *P* values are presented in each box and represent results of comparisons between groups listed on corresponding vertical versus horizontal axes. The colour of each box represents the degree of statistical significance in the difference between groups, with red indicating a highly significant difference, blue a non-significant difference and white borderline (trend) or moderately significant difference, with gradations in between. FG = frontal grey matter; FW = frontal white matter; TG = temporal grey matter; TW = temporal white matter

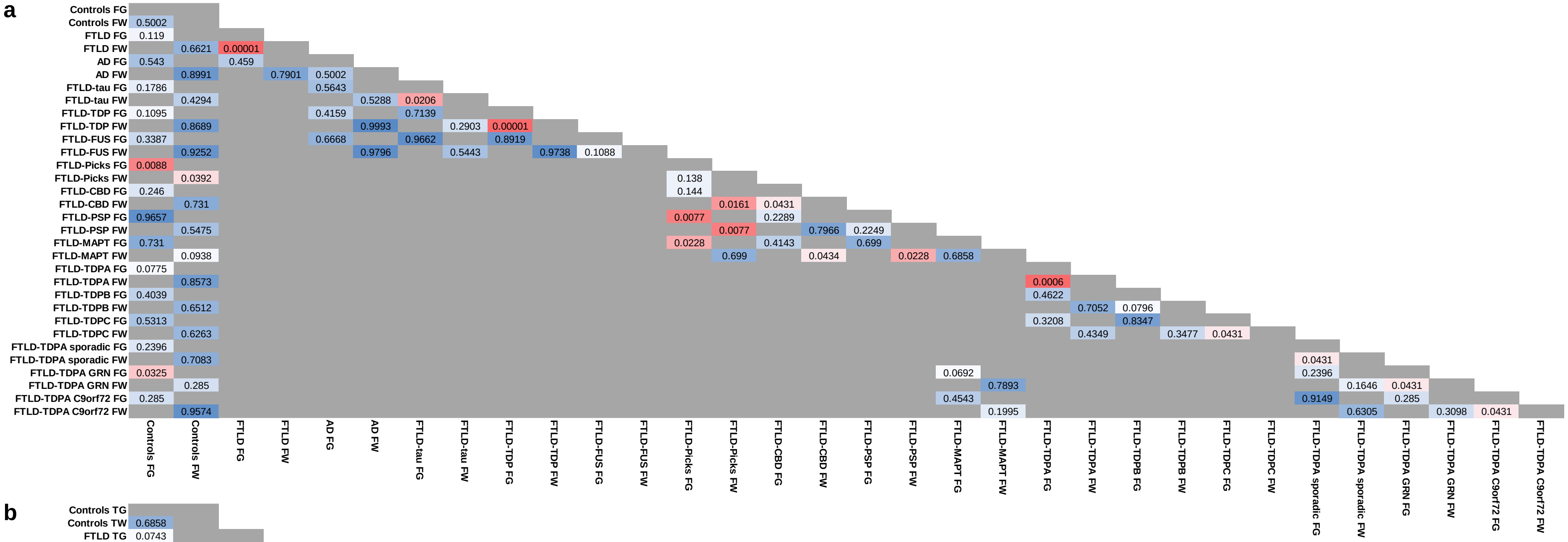

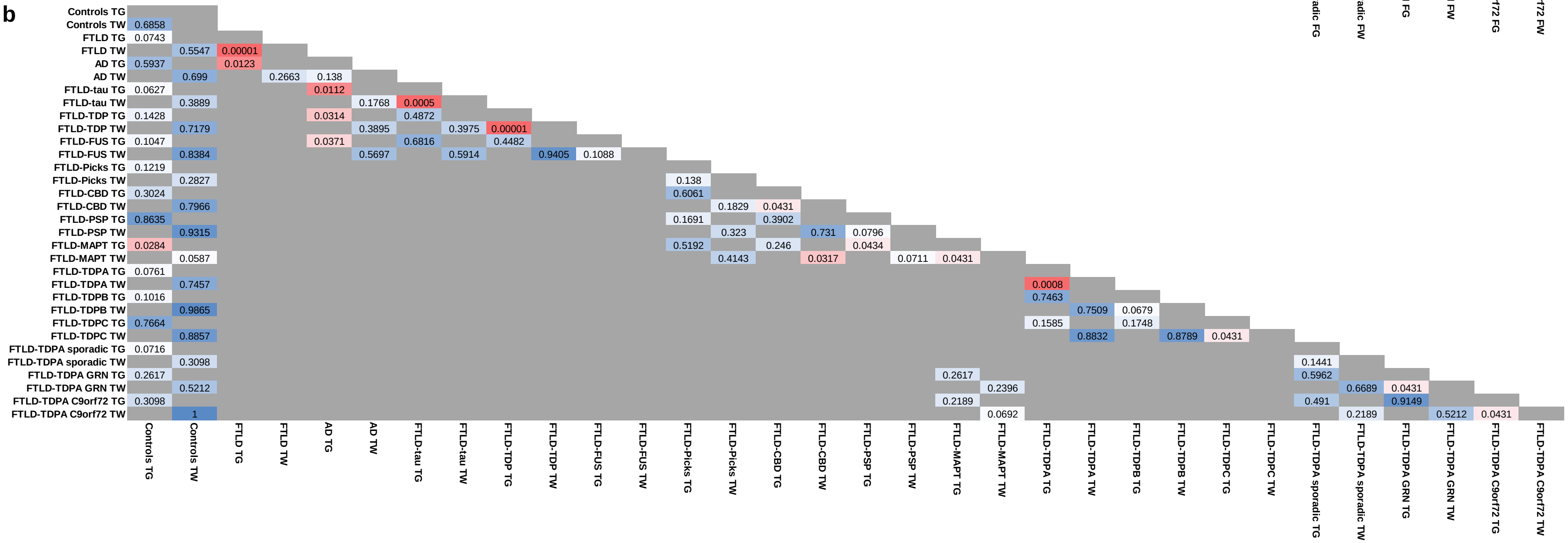

Supplementary Fig. 5

c

	Controls FG	Controls FW	FTLD FG	FTLD FW	AD FG	AD FW	FTLD-tau FG	FTLD-tau FW	FTLD-TDP FG	FTLD-TDP FW	FTLD-FUS FG	FTLD-FUS FW	FTLD-Picks FG	FTLD-Picks FW	FTLD-CBD FG	FTLD-CBD FW	FTLD-PSP FG	FTLD-PSP FW	FTLD-MAPT FG	FTLD-MAPT FW	FTLD-TDPA FG	FTLD-TDPA FW	FTLD-TDPB FG	FTLD-TDPB FW	FTLD-TDPC FG	FTLD-TDPC FW	FTLD-TDPA sporadic FG	FLTD-TDPA sporadic FW	FTLD-TDPA GRN FG	FTLD-TDPA GRN FW	FTLD-TDPA C9orf72 FG	FTLD-TDPA C9orf72 FW
Controls FG																																
Controls FW	0.2249																															
FTLD FG	0.7345																															
FTLD FW		0.4736	0.00001																													
AD FG	0.4652		0.5198																													
AD FW		0.8222		0.3081	0.6858																											
FTLD-tau FG	0.9151				0.4138																											
FTLD-tau FW		0.859				0.9151	0.0124																									
FTLD-TDP FG	0.6638				0.6136		0.5963																									
FTLD-TDP FW		0.1868				0.1076		0.0148	0.0001																							
FTLD-FUS FG	0.5872)				0.8842		0.5703		0.7805																							
FTLD-FUS FW		0.3774				0.2737		0.2137		0.9163																						
FTLD-Picks FG	0.4918																															
FTLD-Picks FW		0.731											0.3452																			
FTLD-CBD FG	0.144												0.0317																			
FTLD-CBD FW		0.8974												0.6365	0.0431																	
FTLD-PSP FG	0.5475												0.9315		0.0392																	
FTLD-PSP FW		0.4393												0.2639		0.5192	0.6858															
FTLD-MAPT FG	0.4143												0.8974		0.0228		0.8299															
FTLD-MAPT FW		0.731												1		0.6365		0.2639	0.3452													
FTLD-TDPA FG	0.6653																															
FTLD-TDPA FW		0.3562																			0.0027											
FTLD-TDPB FG	0.9743																				0.7528											
FTLD-TDPB FW		0.1175																				0.2866	0.1088									
FTLD-TDPC FG	0.8819																				0.8031		0.9231									
FTLD-TDPC FW		0.2067																				0.5247		0.6371	0.0796							
FTLD-TDPA sporadic FG	0.1814																															
FLTD-TDPA sporadic FW		0.0543																									0.138					
FTLD-TDPA GRN FG	0.593																		0.1646								0.0777					
FTLD-TDPA GRN FW		0.7484																		0.9149								0.1345	0.0796			
FTLD-TDPA C9orf72 FG	0.8307																		0.5212								0.285		0.4543			
FTLD-TDPA C9orf72 FW		0.6689																		0.8307								0.1088		0.9149	0.0796	

d

	Controls TG	Controls TW	FTLD TG	FTLD TW	AD TG	AD TW	FTLD-tau TG	FTLD-tau TW	FTLD-TDP TG	FTLD-TDP TW	FTLD-FUS TG	FTLD-FUS TW	FTLD-Picks TG	FTLD-Picks TW	FTLD-CBD TG	FTLD-CBD TW	FTLD-PSP TG	FTLD-PSP TW	FTLD-MAPT TG	FTLD-MAPT TW	FTLD-TDPA TG	FTLD-TDPA TW	FTLD-TDPB TG	FTLD-TDPB TW	FTLD-TDPC TG	FTLD-TDPC TW	FTLD-TDPA sporadic TG	FTLD-TDPA sporadic TW	FTLD-TDPA GRN TG	FTLD-TDPA GRN TW	FTLD-TDPA C9orf72 TG	FTLD-TDPA C9orf72 TW
Controls TG																																
Controls TW	0.2249																															
FTLD TG	0.7222																															
FTLD TW		0.5362	0.00001																													
AD TG	0.2943		0.291																													
AD TW		0.6941		0.2511	0.2249																											
FTLD-tau TG	0.7944				0.2865																											
FTLD-tau TW		0.5458				0.2707	0.0032																									
FTLD-TDP TG	0.6328				0.3836		0.7297																									
FTLD-TDP TW		0.6414				0.3312		0.8094	0.0152																							
FTLD-FUS TG	0.9332				0.2835		0.7334		0.5898																							
FTLD-FUS TW		0.3679				0.2037		0.5814		0.4874	0.0679																					
FTLD-Picks TG	0.6061																															
FTLD-Picks TW		0.3445											0.2249																			
FTLD-CBD TG	0.4143												0.1829																			
FTLD-CBD TW		0.8299												0.4651	0.0431																	
FTLD-PSP TG	0.8299												0.7636		0.3024																	
FTLD-PSP TW		0.6674												0.6061		0.8299	0.0796															
FTLD-MAPT TG	0.6061												1		0.1829		0.7636															
FTLD-MAPT TW		0.4393												0.8635		0.5765		0.731	0.2249													
FTLD-TDPA TG	0.608																															
FTLD-TDPA TW		0.6221																			0.0262											
FTLD-TDPB TG	0.2984																				0.4298											
FTLD-TDPB TW		0.8219																				0.8887	1									
FTLD-TDPC TG	0.9113																				0.5154		0.2558									
FTLD-TDPC TW		0.9408																				0.6883		0.8722	0.138							
FTLD-TDPA sporadic TG	0.1995																															
FTLD-TDPA sporadic TW		0.4227																									0.5002					
FTLD-TDPA GRN TG	0.9574																		0.3924								0.1814					
FTLD-TDPA GRN TW		0.9149																		0.4227								0.3635	0.2249			
FTLD-TDPA C9orf72 TG	0.8307																		0.5565								0.2617		0.7893			
FTLD-TDPA C9orf72 TW		0.7083																		0.593								0.593		0.7893	0.0431	

Supplementary Fig. 5

e

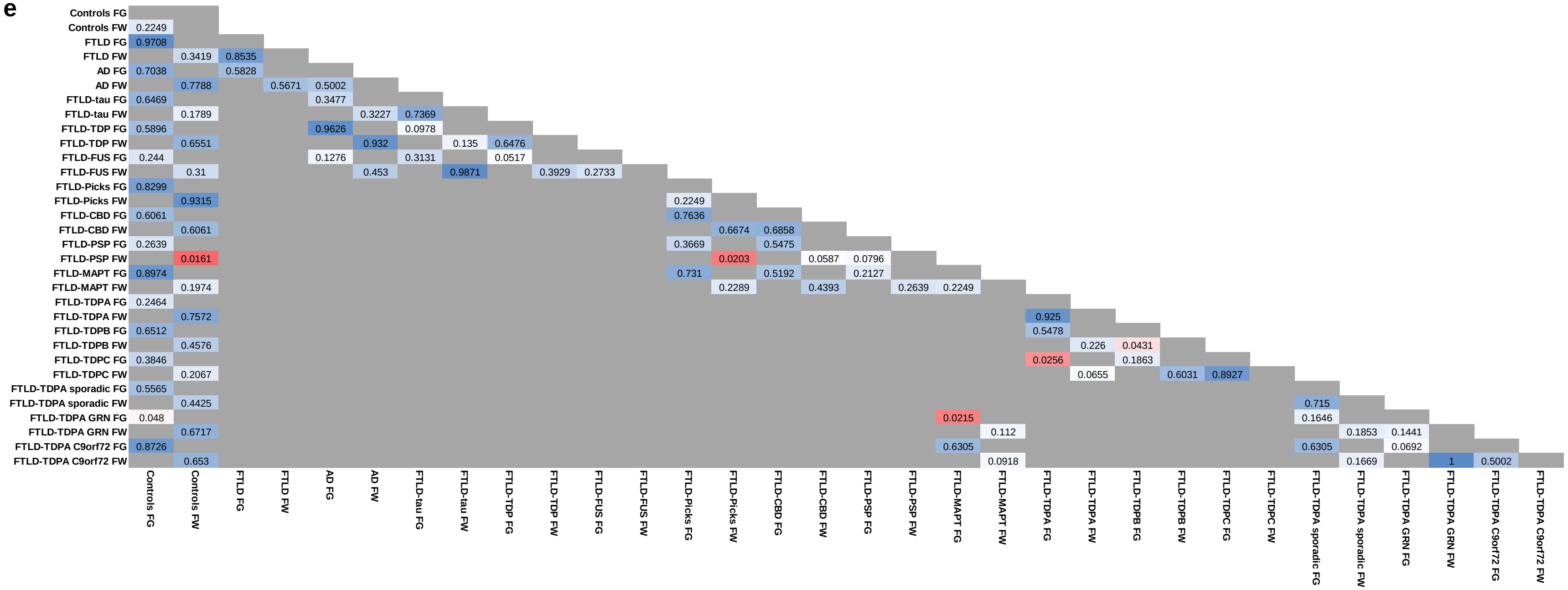

f

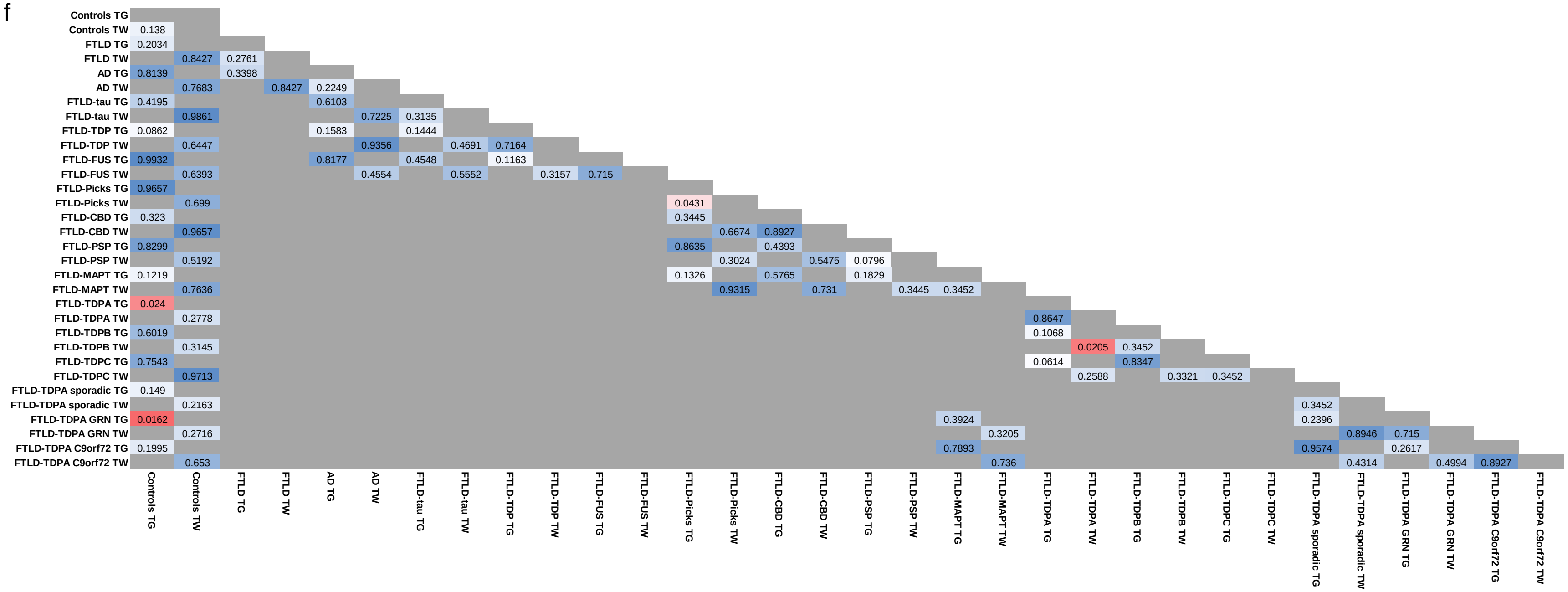

Supplementary Fig. 6 Heat map of comparisons of microglial perimeter between all groups and between grey and white matter within each group. a and **b:** CD68-positive microglia; **c** and **d:** CR3/43-positive microglia; **e** and **f:** Iba1-positive microglia. Frontal lobe: **a, c, e**. Temporal lobe: **b, d, f.** *P* values are presented in each box and represent results of comparisons between groups listed on corresponding vertical versus horizontal axes. The colour of each box represents the degree of statistical significance in the difference between groups, with red indicating a highly significant difference, blue a non-significant difference and white borderline (trend) or moderately significant difference, with gradations in between. FG = frontal grey matter; FW = frontal white matter; TG = temporal grey matter; TW = temporal white matter

a

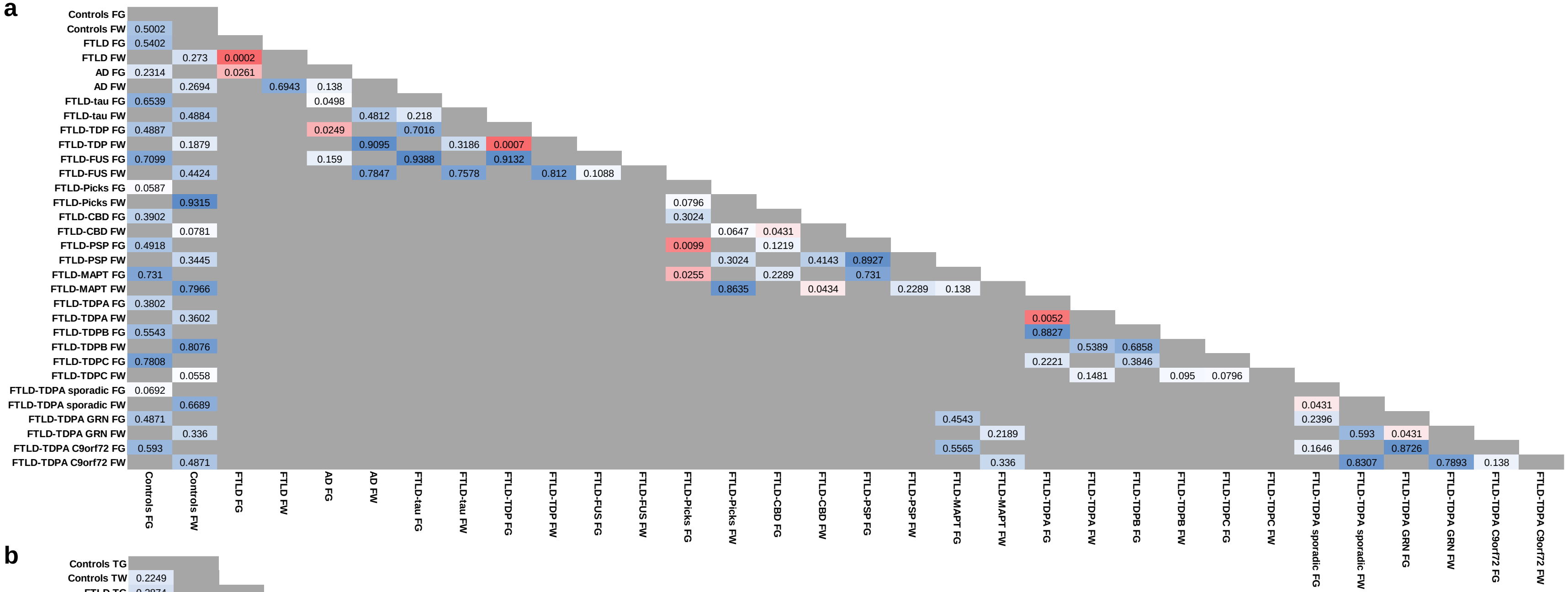

b

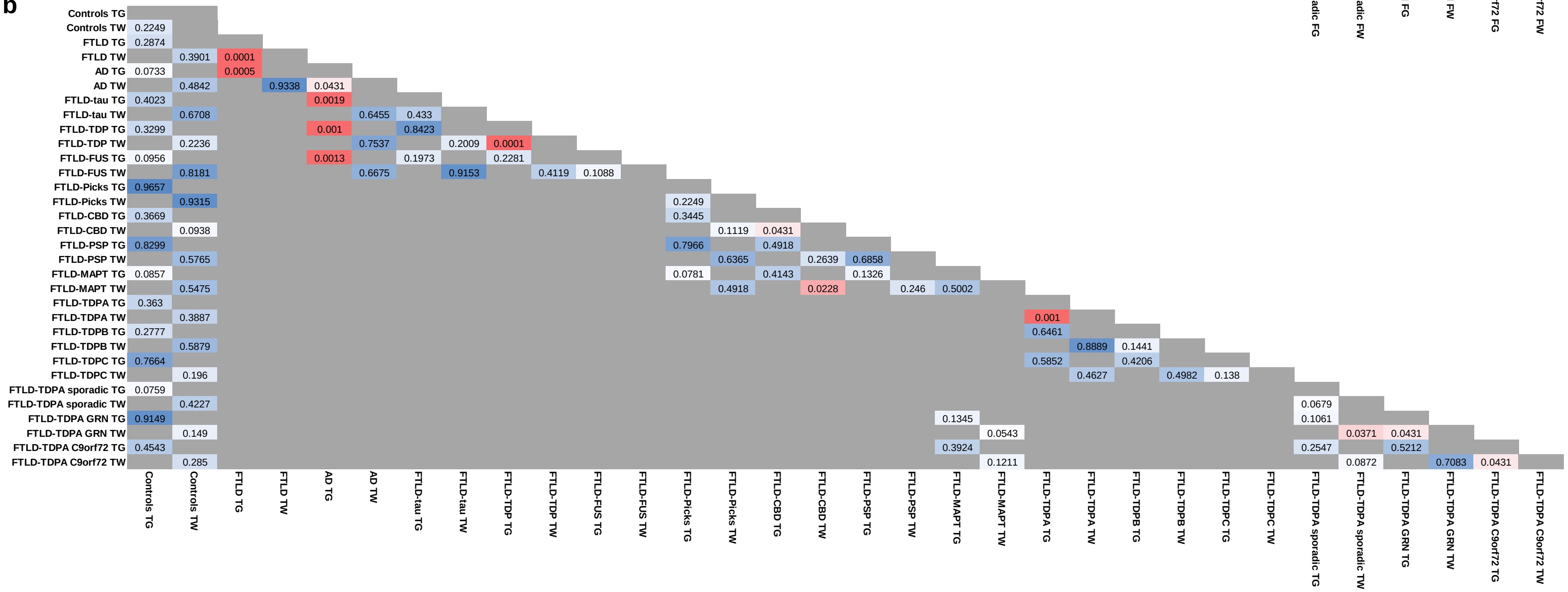

Supplementary Fig. 6

c

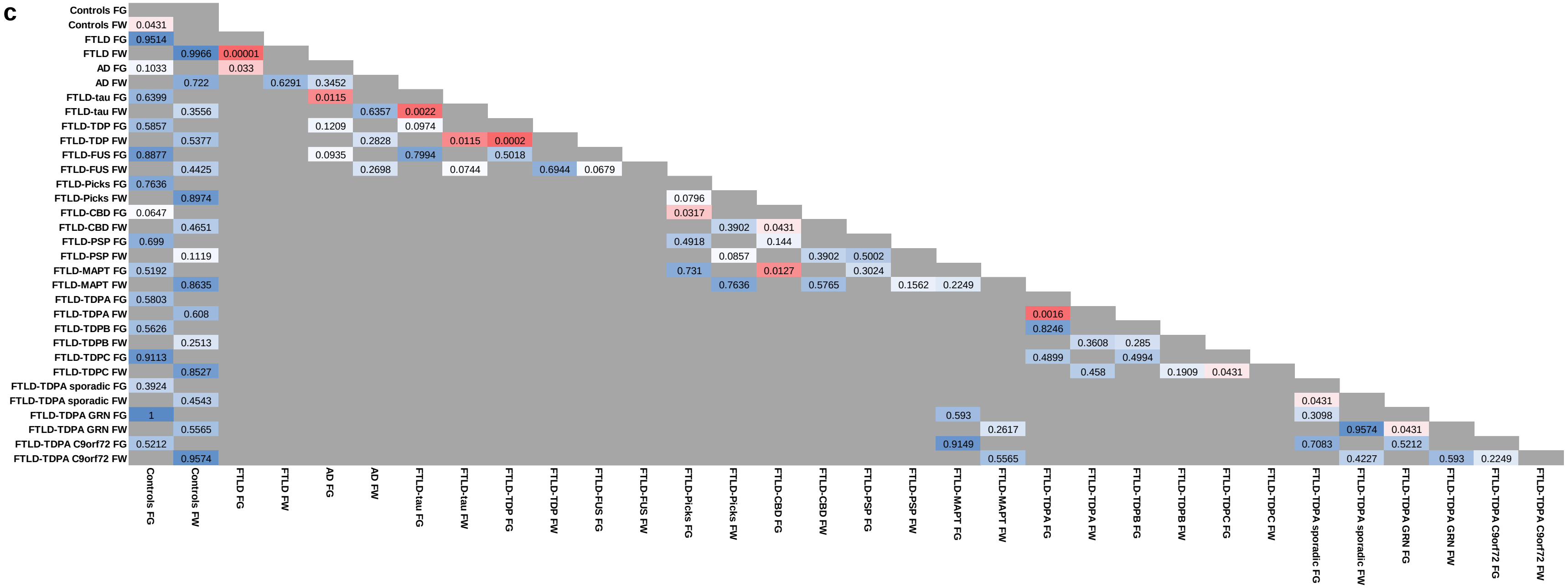

d

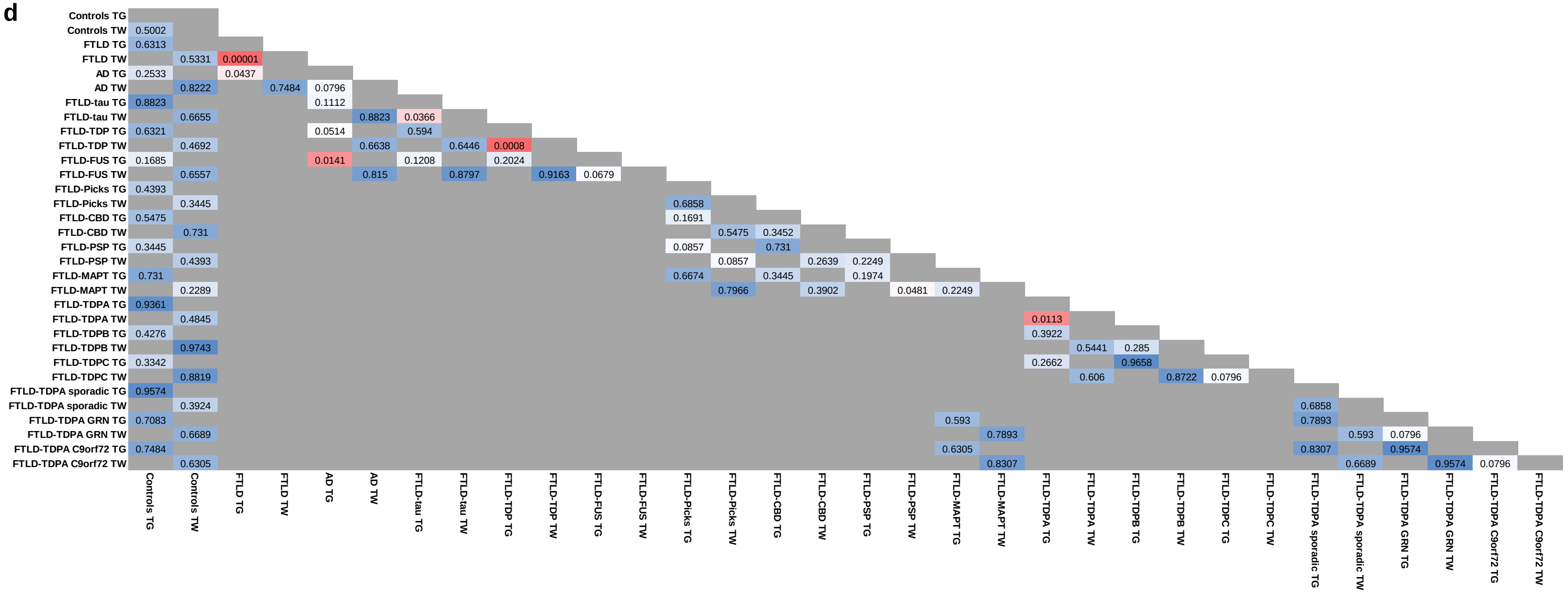

Supplementary Fig. 6

e

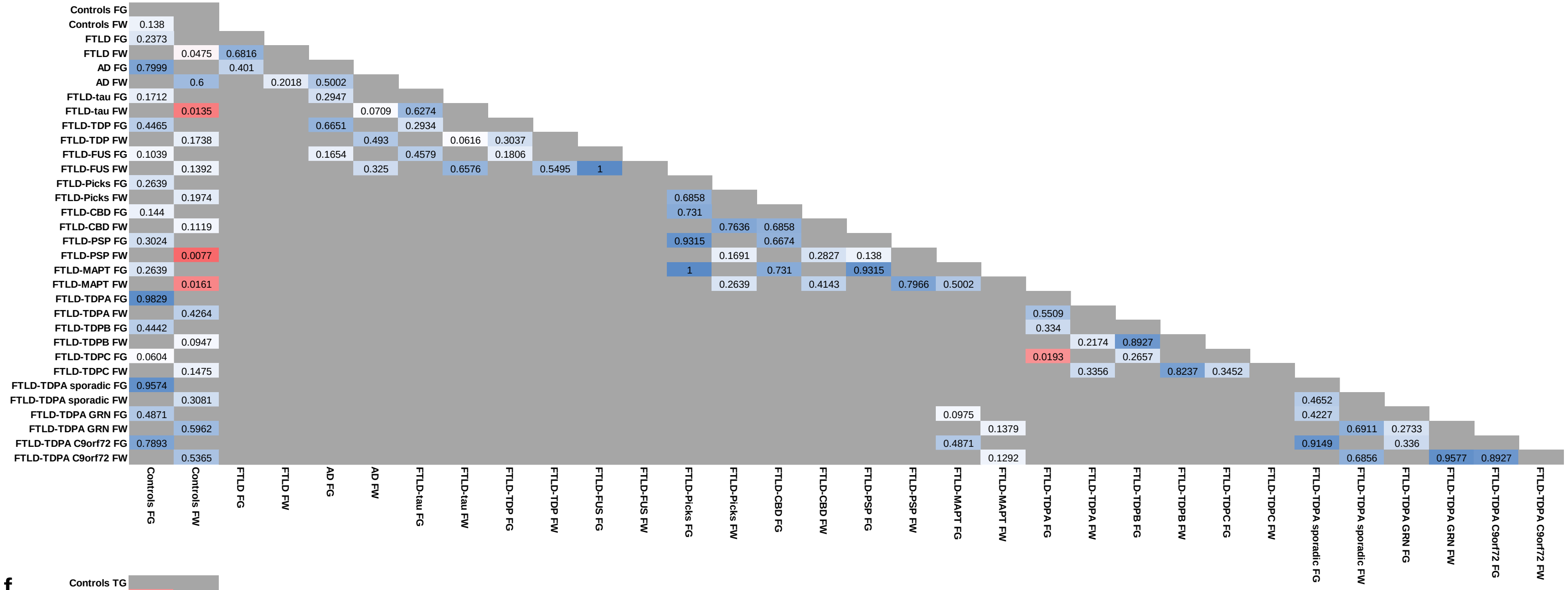

f

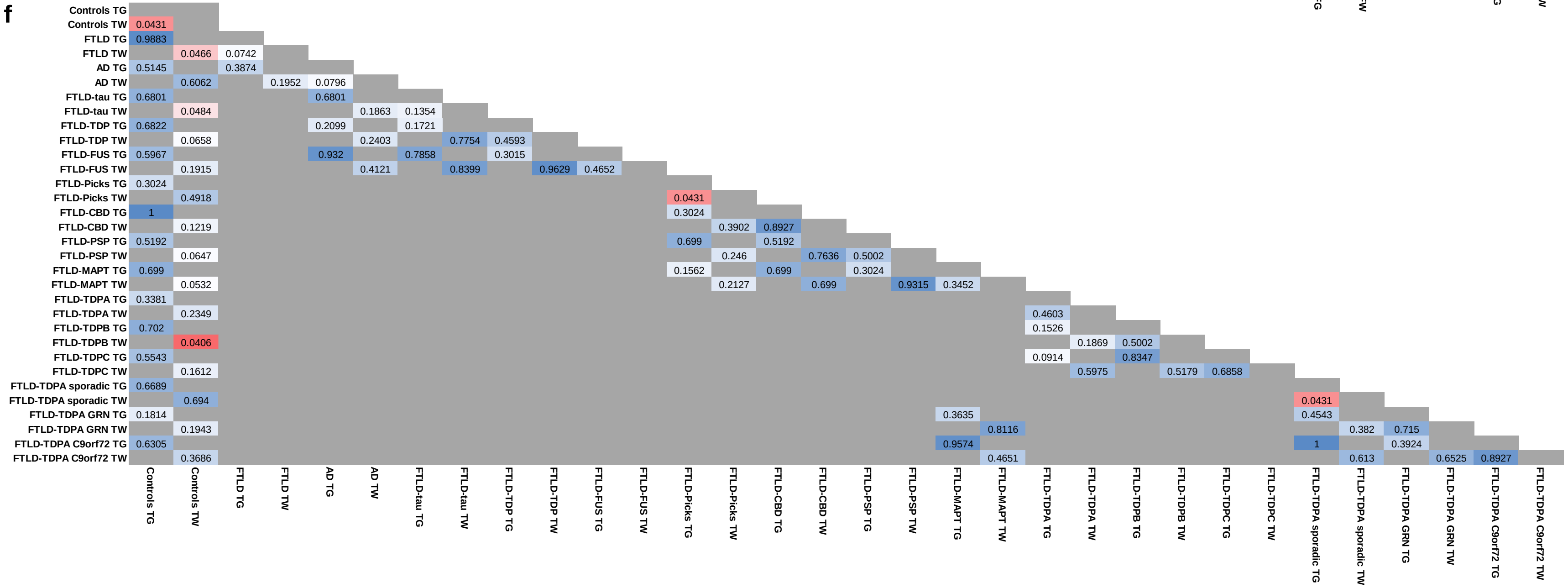

Supplementary Fig. 7 Heat map of comparisons of microglial dystrophy scores between all groups and between grey and white matter within each group. a frontal lobe; **b** temporal lobe. *P* values are presented in each box and represent results of comparisons between groups listed on corresponding vertical versus horizontal axes. The colour of each box represents the degree of statistical significance in the difference between groups, with red indicating a highly significant difference, blue a non-significant difference and white borderline (trend) or moderately significant difference, with gradations in between. FG = frontal grey matter; FW = frontal white matter; TG = temporal grey matter; TW = temporal white matter

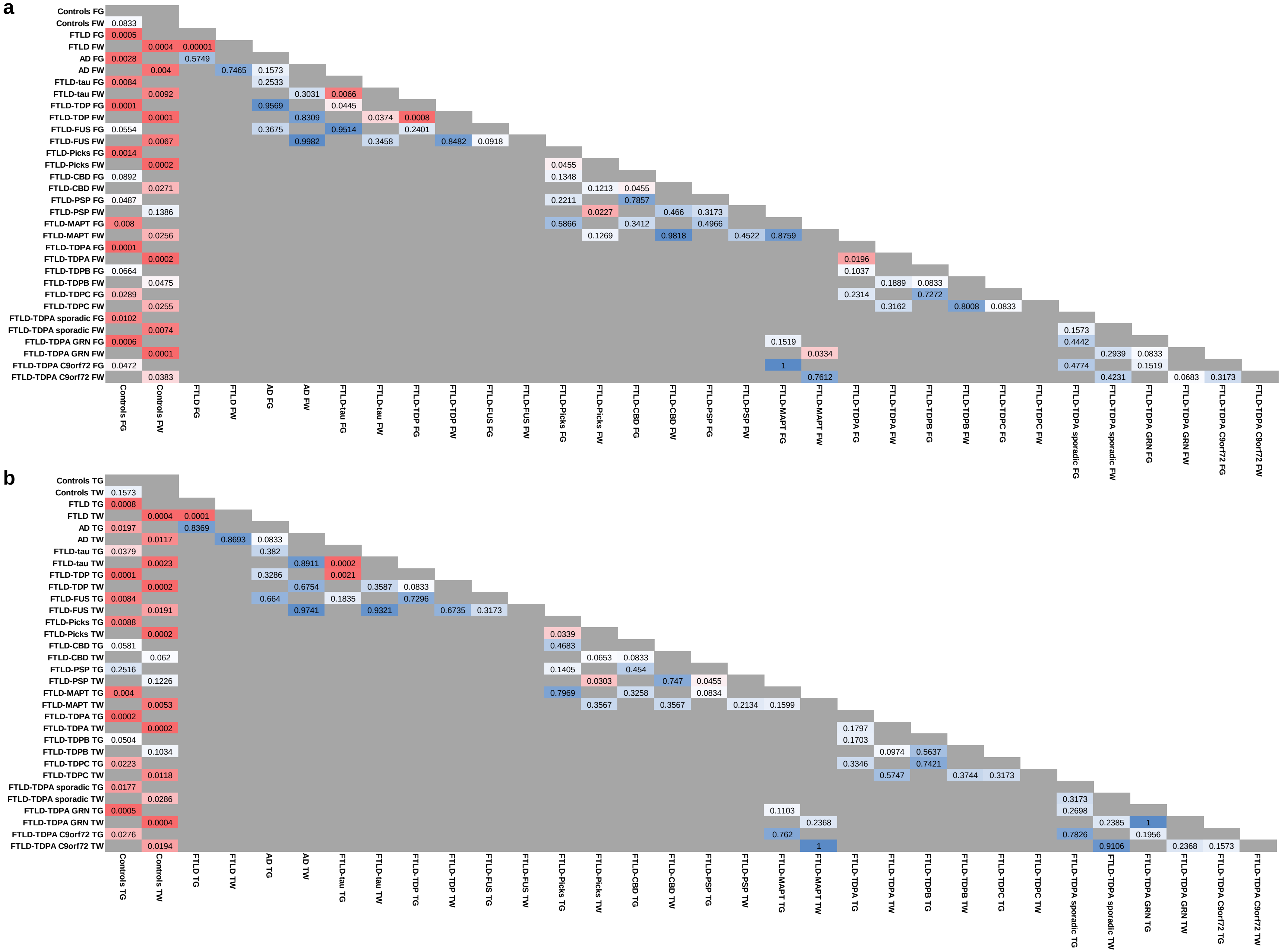

Supplement: Supplementary file 2 — Additional file 2: Supplementary Fig. 4. Heat map of comparisons of microglial burden between all groups and between grey and white matter within each group. a and b: CD68-positive microglia; c and d: CR3/43-positive microglia; e and f: Iba1-positive microglia. Frontal lobe: a, c, e. Temporal lobe: b, d, f. P values are presented in each box and represent results of comparisons between groups listed on corresponding vertical versus horizontal axes. The colour of each box represents the degree of statistical significance in the difference between groups, with red indicating a highly significant difference, blue a non-significant difference and white borderline (trend) or moderately significant difference, with gradations in between. FG = frontal grey matter; FW = frontal white matter; TG = temporal grey matter; TW = temporal white matter. Supplementary Fig. 5. Heat map of comparisons of microglial circularity between all groups and between grey and white matter within each group. a and b: CD68-positive microglia; c and d: CR3/43-positive microglia; e and f: Iba1-positive microglia. Frontal lobe: a, c, e. Temporal lobe: b, d, f. P values are presented in each box and represent results of comparisons between groups listed on corresponding vertical versus horizontal axes. The colour of each box represents the degree of statistical significance in the difference between groups, with red indicating a highly significant difference, blue a non-significant difference and white borderline (trend) or moderately significant difference, with gradations in between. FG = frontal grey matter; FW = frontal white matter; TG = temporal grey matter; TW = temporal white matter. Supplementary Fig. 6. Heat map of comparisons of microglial perimeter between all groups and between grey and white matter within each group. a and b: CD68-positive microglia; c and d: CR3/43-positive microglia; e and f: Iba1-positive microglia. Frontal lobe: a, c, e. Temporal lobe: b, d, f. P values are presente [file 12974_2020_1907_MOESM2_ESM.pdf]
